# Comprehensive transcriptomic and proteomic analyses of antroquinonol biosynthetic genes and enzymes in *Antrodia camphorata*

**DOI:** 10.1186/s13568-020-01076-6

**Published:** 2020-08-03

**Authors:** Xiaofeng Liu, Yongjun Xia, Yao Zhang, Caiyun Yang, Zhiqiang Xiong, Xin Song, Lianzhong Ai

**Affiliations:** Shanghai Engineering Research Center of Food Microbiology, School of Medical Instrument and Food Engineering, University of Shanghai for Science and Technology, 516 Jungong Road, Shanghai, 200093 People’s Republic of China

**Keywords:** *Antrodia camphorata*, Antroquinonol, Transcriptome, iTRAQ, Q-PCR

## Abstract

Antroquinonol (AQ) has several remarkable bioactivities in acute myeloid leukaemia and pancreatic cancer, but difficulties in the mass production of AQ hamper its applications. Currently, molecular biotechnology methods, such as gene overexpression, have been widely used to increase the production of metabolites. However, AQ biosynthetic genes and enzymes are poorly understood. In this study, an integrated study coupling RNA-Seq and isobaric tags for relative and absolute quantitation (iTRAQ) were used to identify AQ synthesis-related genes and enzymes in *Antrodia camphorata* during coenzyme Q_0_-induced fermentation (FM). The upregulated genes related to acetyl-CoA synthesis indicated that acetyl-CoA enters the mevalonate pathway to form the farnesyl tail precursor of AQ. The *metE* gene for an enzyme with methyl transfer activity provided sufficient methyl groups for AQ structure formation. The *CoQ2* and *ubiA* genes encode *p*-hydroxybenzoate polyprenyl transferase, linking coenzyme Q_0_ and the polyisoprene side chain to form coenzyme Q_3_. NADH is transformed into NAD+ and releases two electrons, which may be beneficial for the conversion of coenzyme Q_3_ to AQ. Understanding the biosynthetic genes and enzymes of AQ is important for improving its production by genetic means in the future.

## Key points

1. RNA-Seq and iTRAQ were used to identify AQ synthesis-related genes and enzymes.
2. Ubiquinone and other terpenoid-quinone biosynthesis pathway was upregulated.
3. Genes such as *metE*, *CoQ2*, and *ubiA* are important for AQ production.

## Introduction

*Antrodia camphorata*, a unique basidiomycete, is indigenous species of Taiwan. It has been used as a traditional medicine for treat diverse discomforts such as abdominal pain, hangover, and diarrhea (Wu et al. 1997). The fruiting body of *A. camphorata* is rich in bioactive metabolites, but the fruiting body can only grow on the rare *Cinnamomum kanehirae Hayata* at an extremely slow rate. The mycelium of *A. camphorata* can easily be cultured on a large scale via submerged fermentation, but it has been found to be deficient in specifically bioactive metabolites, such as antroquinonol (AQ) (Lu et al. 2013). AQ, an ubiquinone derivative, was identified from the solid-state fermented mycelium of *A. camphorata* in 2007 (Lee et al. 2007). The remarkably therapeutic activities of AQ in many diseases have been documented, including liver and kidney diseases (Angamuthu et al. 2019), *Alzheimer*’*s* disease (Chang et al. 2015), and cancer (Chiang et al. 2010).

In a previous study, it is successful to stimulate the biosynthesis of AQ in submerged fermentation by addition of precursor coenzyme Q_0_ (Xia et al. 2018). Therefore, we hypothesize that the addition of coenzyme Q_0_ would stimulate the expression of genes involved in AQ synthesis. However, a lack research on the molecular genetics of related metabolites in *A. camphorata*, such as transcriptomic and proteomic resources, currently hinders such studies on AQ biosynthetic genes and enzymes. Large-scale sequencing techniques have been widely used, which improve the efficiency of understanding the differential gene expression patterns within microorganism (MacLean et al. 2009; Juan et al. 2010).

In the present study, we focused on the gene transcriptional and protein expression features of *A. camphorata* S-29 in submerged fermentation with and without the precursor coenzyme Q_0_ using second-generation sequencing on the Illumina HiSeq™ 2000 platform. We further validated functional genes associated with AQ synthesis through quantitative polymerase chain reaction (q-PCR). Two “omics” levels of analyses indicated that the formation of AQ constitutes a highly complicated and genetically programmed process that requires the participation of multiple regulators. This investigation advances our understanding of genes and enzymes involved in AQ synthesis during FM.

## Materials and methods

### Microorganism and cultivation

The microorganism used in this study was *A. camphorata* S-29, which was deposited in the China General Microbiological Culture Collection Center(CGMCC No. 9590). Seed medium was perpared according to the method of Xia et al. (2018).

The fermentation medium was prepared according to our previous report (Liu et al. 2020). For the investigation on the regulatory mechanisms, 0.3 g/L coenzyme Q_0_ was filtered and added into the fermentation broth. The experiments were carried out with three biological repeats. Mycelia in the fermentation broth were collected by centrifugation at 3000*g* for 10 min at 4 °C. The mycelia sediment was washed twice with 40 mL of potassium phosphate buffer, rapidly frozen in liquid nitrogen, and stored at − 80 °C for further use.

The absence of precursor (coenzyme Q_0_) during fermentation was regarded as conventional submerged fermentation (KB). Adding coenzyme Q_0_ during fermentation was named as coenzyme Q_0_-induced fermentation (FM). KB4 (or FM4), KB5 (or FM5), and KB10 (or FM10) representing the mycelia of *A. camphorata* S-29 were collected on days 4, 5, and 10, respectively.

### Differential transcriptome analysis

The days 4, 5, and 10 samples were sequenced by Shanghai Majorbio Biopharm Technology Co., Ltd (Shanghai, China), with the Illumina HiSeq™ 2000 platform (Bai et al. 2015). The raw data were filtered by removing the low quality reads, sequences of length less than 20 bp, and reads containing adapters to obtain clean data. A rapid comparison of the sequencing data with a reference genome was performed by Tophat2 software, which can also be used to detect events such as variable shear and gene fusion (Trapnell et al. 2012). Analyses of sequence saturation, gene coverage, and duplicate reads in the transcriptome were performed using RSeQC-2.6.3 software (Vera Alvarez et al. 2019). Analysis of gene expression was performed using RSEM software (Li and Dewey 2011). Furthermore, edgeR software was used to calculate the differential expression based on gene read count data (Robinson et al. 2010). The differentially expressed genes (DEGs) were classified by the Gene Ontology (GO, http://geneontology.org/) database according to the biological process, cellular components, and molecular functions. KOBAS software (Peking University, Beijing, China) was used to test the enrichment of the DEGs; in particular, the Kyoto Encyclopedia of Genes and Genomes (KEGG, http://www.genome.jp/kegg/) pathways (Xie et al. 2011).

### Protein quantification by isobaric tags for relative and absolute quantitation (iTRAQ)

Protein quantification by iTRAQ was carried out according to the method of Lu et al. (2017). The mixed peptides were sequenced by Majorbio Biopharm Technology Co., Ltd (Shanghai, China). All identified proteins were annotated by GO and KEGG. Differential proteins were screened based on differential multiples and *P*-values. The data were analysed by the Majorbio I-Sanger Cloud Platform (https://cloud.majorbio.com/).

The enrichment ratio (ER) was calculated as follow: ER = CN/BN, where CN is the number of proteins (or genes), which were enriched to this KEGG in the protein (or gene) set, and BN is the number of proteins (or genes), which were enriched to this KEGG among all annotated proteins (or genes).

### Validation of AQ synthesis-related genes using q-PCR

Reverse transcription of cDNA was performed according to the manufacturer’s instructions (PrimeScript 1st strand cDNA synthesis kit, Takara, Japan). Q-PCR was performed using LightCycler^®^ 96 system (Roche, Switzerland) detection. The *18S rRNA* gene of *A. camphorata* S-29 was used as the internal standard. Primers used in this research for q-PCR (Additional file 1: Table S1) were designed by Oligo 7.0 software. Relative expression level of gene was quantified based on the 2^−∆∆Ct^ method. It was carried out with three biological repeats. Values are given as the means ± standard deviations (n = 3).

### Sequencing data

The FASTQ format raw reads were deposited to the National Center for Biotechnology Information Short Read Archive (NCBI SRA) database (accession: PRJNA543624 and PRJNA622907). The mass spectrometry proteomics data have been deposited to the ProteomeXchange Consortium (http://proteomecentral.proteomexchange.org) via the iProX partner repository with the dataset identifier PXD018004 (Ma et al. 2019).

## Results

### Identification of DEGs via transcriptome sequencing

**Fig. 1:**
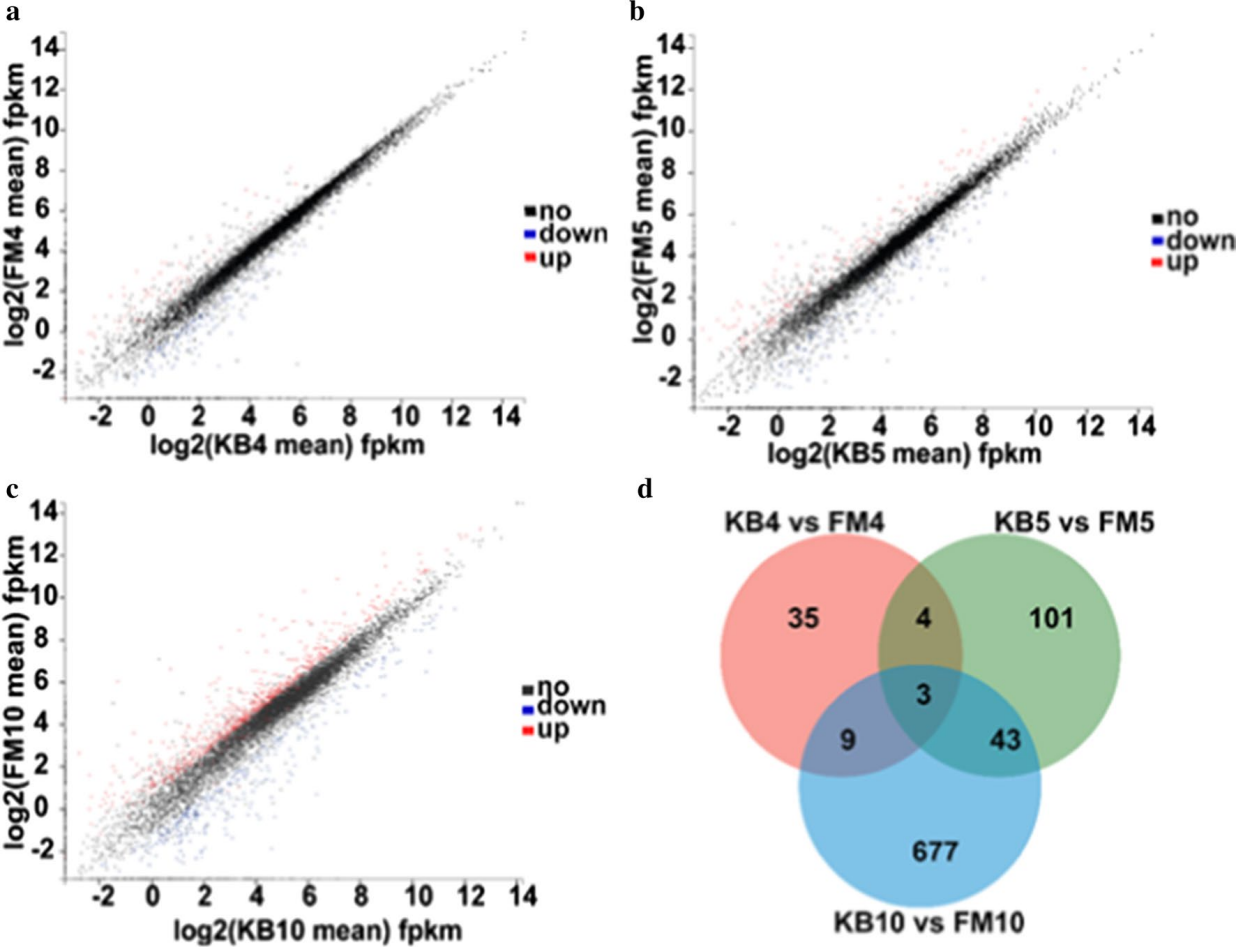
DEGs between KB and FM. **a**–**c** a scatter plot of the DEGs during FM compared with KB on days 4, 5, and 10, respectively. Significantly upregulated or downregulated genes were labeled with red dots or blue dots, respectively. **d** Venn diagram of the DEGs on days 4, 5, and 10, respectively

The highest number of the expressed genes of *A. camphorata* S-29 was from FM5 (7478), followed by KB5 (7470), KB4 (7456), FM4 (7436), KB10 (7409), and FM10 (7356), respectively (Additional file 1: Figure S1). KEGG map shows that the highest number of genes were involved in metabolism. Over 200 genes annotated to the KEGG functional categories of translation, carbohydrate metabolism, folding, sorting and degradation, and amino acid metabolism (Additional file 1: Figure S2). Scatter plots and Venn diagram were used to analyse of DEGs between KB and FM (Fig. 1). As shown in Fig. 1a–c, the DEGs between KB and FM increased gradually from 4 to 10 days. The number of DEGs were 51, 151, and 732 on days 4, 5, and 10, respectively (Fig. 1d). A total of 35 DEGs were only expressed between KB4 and FM4, with 101 DEGs expressed between KB5 and FM5. In comparison, 677 DEGs were only expressed between KB10 and FM10.

### Functional classification of genes by GO and KEGG analysis

**Fig. 2:**
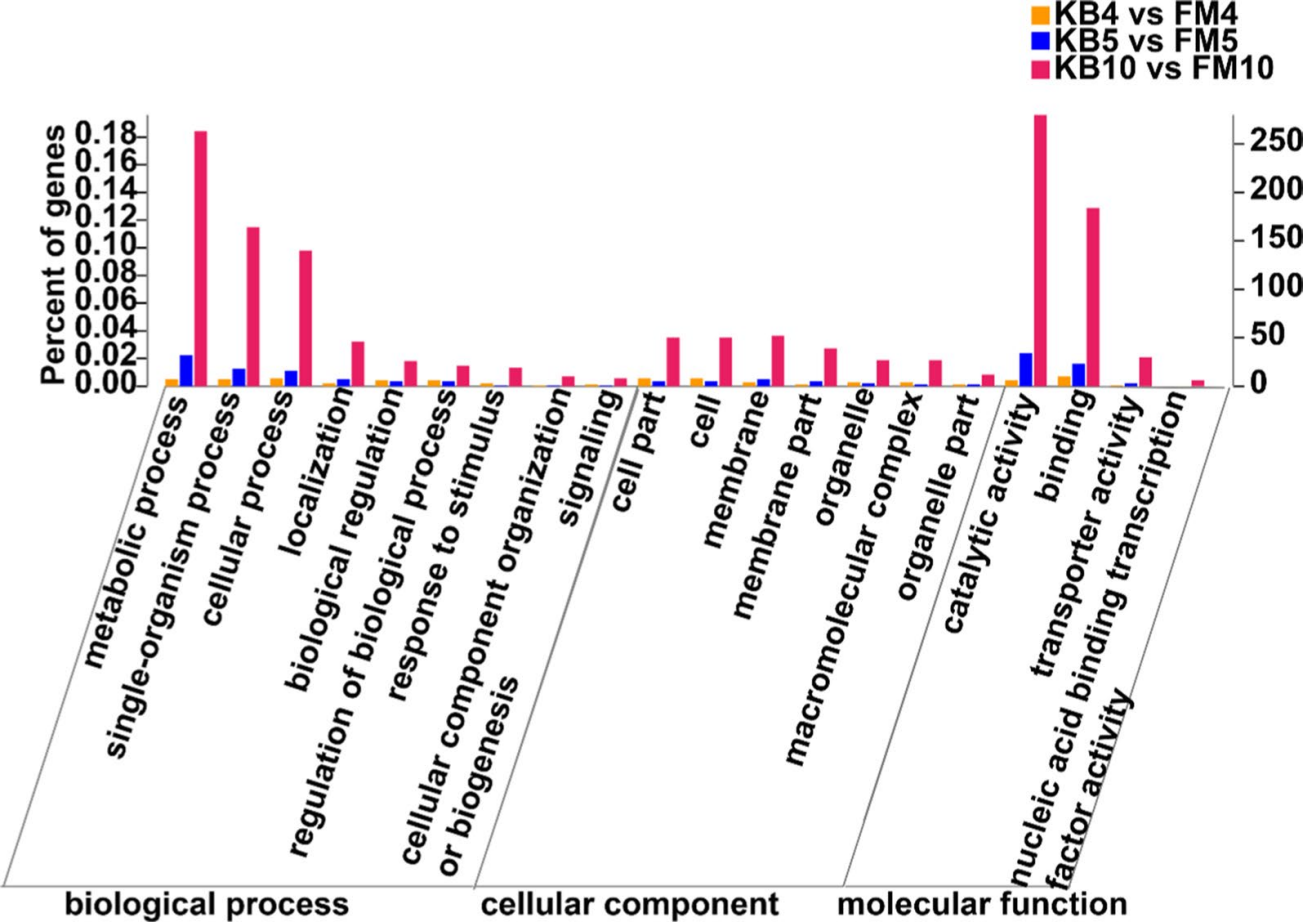
GO annotation of DEGs. Each bar represent the percentage and number of genes in each GO category. Yellow, blue, and red colouring represent DEGs on days 4, 5, and 10, respectively

These genes were classified into 20 GO categories, and all GO categories were then assigned into three main categories: biological process, cellular component, and molecular function. As shown in Fig. 2, in the biological processes, 9 significant enrichment items were found. Particularly, a large number of genes involved in metabolic process, single-organism metabolic process, and cellular processes were significantly enriched in FM10 compared to KB10. In the cellular component, 7 significant enrichment items were found. In the molecular functionality, 4 significant enrichment items, especially catalytic activity and binding, were identified in FM10 compared to KB10.

**Fig. 3:**
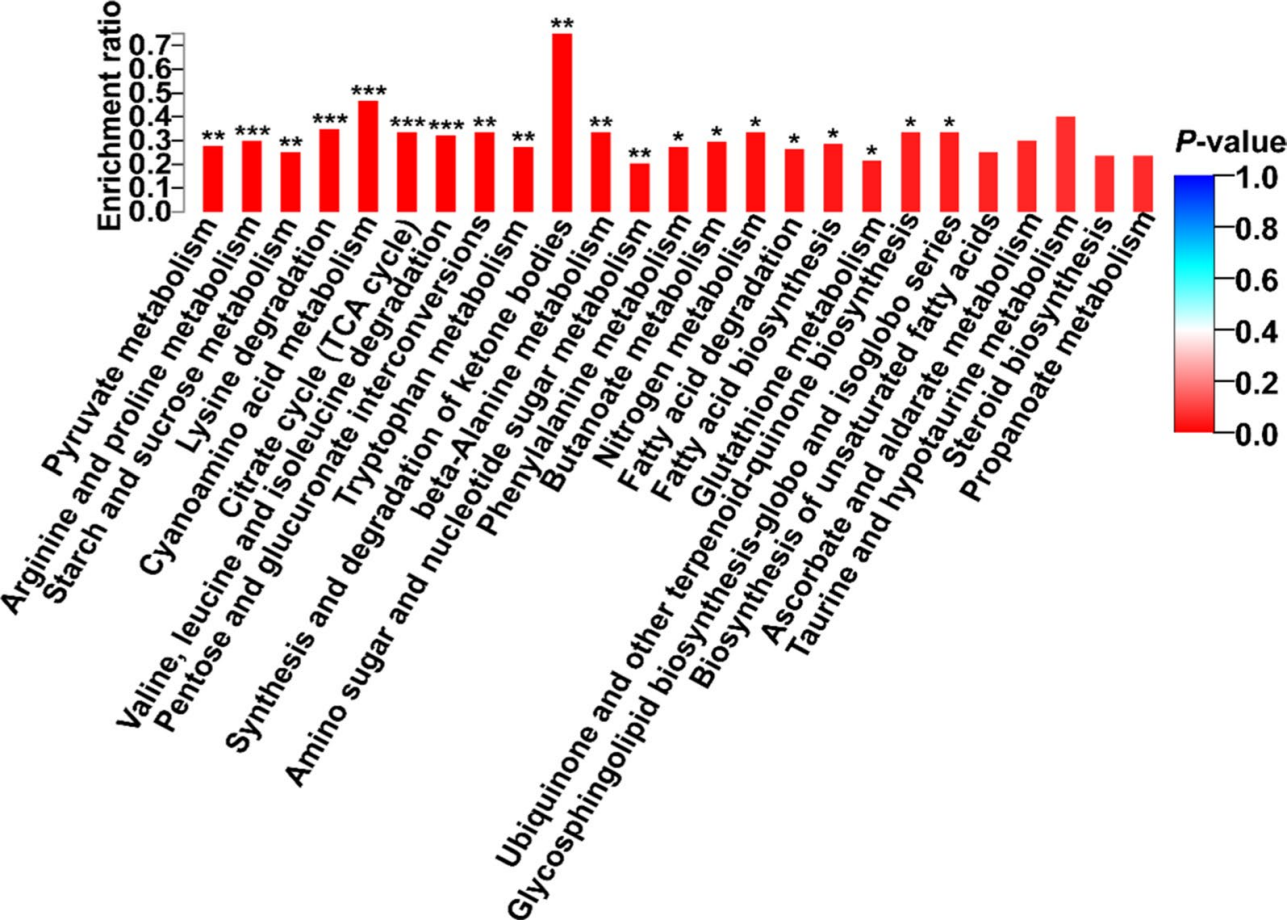
Analysis of the DEGs between KB10 and FM10 based on the KEGG enrichment map. *P < 0.05, **P < 0.01, ***P < 0.001

In the early phase of coenzyme Q_0_ addition (on day 4), only the SNARE interactions in vesicular transport pathway was significantly enriched in FM4 compared to KB4 (Additional file 1: Figure S3A, *P* < 0.05). The arginine and proline metabolism, tryptophan metabolism, and C5-branched dibasic acid metabolism were significantly enriched in FM5 compared to KB5 (Additional file 1: Figure S3B, *P* < 0.05). As shown in Fig. 3, 20 metabolic pathways were significantly enriched on day 10 (*P* < 0.05). Arginine and proline metabolism, lysine degradation, cyanoamino acid metabolism, citrate cycle (TCA cycle), and valine, leucine and isoleucine degradation pathways were significantly enriched (*P* < 0.001). Amino sugar and nucleotide sugar metabolism pathway had the largest number of enriched the DEGs among 20 most significant enrichment pathways (Table 1). The genes of *atoB*, *ALDH*, and *ECHS1* participated in lysine degradation, valine, leucine and isoleucine degradation, tryptophan metabolism, and fatty acid degradation pathways at the same time. It is noteworthy that the DEGs of ubiquinone and other terpenoid-quinone biosynthesis pathway were *CoQ2* and *CoQ5* (Table 1). Additional file 1: Table S2 shows that the *CoQ2* gene, which encodes 4-hydroxybenzoate polyprenyltransferase, was upregulated, while the *CoQ5* gene, which encodes 2-methoxy-6-polyprenyl-1,4-benzoquinol methylase, was downregulated on day 10.

**Table 1:** Annotations of the KEGG pathway for DEGs on day 10

Pathway	Pathway ID	Ratio in study	*P*-value	Genes
Pyruvate metabolism	map00620	8/190	0.00112	*lldD*; *IMS*; *ACACA*; *atoB*; *FH*; *ALDH*; *maeB*; *pckA*
Arginine and proline metabolism	map00330	4/190	0.00033	*amiE*; *PRODH*; *ALDH*; *speB*
Starch and sucrose metabolism	map00500	7/190	0.00102	*TPS*; *GPI*; *E3.2.1.58*; *UGP2*; *E2.4.1.34*; *AGL*; *bglX*
Lysine degradation	map00310	6/190	0.00069	*SUV39H*; *OGDH*; *ALDH*; *EZH2*; *ECHS1*; *atoB*
Cyanoamino acid metabolism	map00460	3/190	0.00018	*CD224*; *ggt*; *bglX*
Citrate cycle (TCA cycle)	map00020	7/190	0.00096	*OGDH*; *fumC*; *ACO*; *ACLY*; *SDHA*; *pckA*; *CS*
Valine, leucine and isoleucine degradation	map00280	7/190	0.00062	*E2.3.3.10*; *atoB*; *OXCT*; *IVD*; *ALDH*; *ECHS1*; *HIBADH*
Pentose and glucuronate interconversions	map00040	5/190	0.00202	*XYLB*; *UGP2*; *SORD*; *ALDH*; *ARD1*
Tryptophan metabolism	map00380	5/190	0.00230	*OGDH*; *ALDH*; *ECHS1*; *amiE*; *atoB*
Synthesis and degradation of ketone bodies	map00072	3/190	0.00295	*OXCT*; *E2.3.3.10*; *atoB*
Beta-alanine metabolism	map00410	3/190	0.00427	*AOC3*; *ALDH*; *ECHS1*
Amino sugar and nucleotide sugar metabolism	map00520	9/190	0.00900	*manC*; *HEXA_B*; *GPI*; *E3.2.1.14*; *UGP2*; *E1.6.2.2*; *CHS1*; *nagZ*; *abfA*
Phenylalanine metabolism	map00360	3/190	0.01252	*AOC3*; *PAAH*; *amiE*
Butanoate metabolism	map00650	5/190	0.01611	*OXCT*; *PAAH*; *ECHS1*; *E2.3.3.10*; *atoB*
Nitrogen metabolism	map00910	2/190	0.01972	*NIT*-*6*; *NR*
Fatty acid degradation	map00071	3/190	0.02598	*ALDH*; *ECHS1*; *atoB*
Fatty acid biosynthesis	map00061	4/190	0.03441	*FAS2*; *ACACA*; *fabG*; *FAS1*
Glutathione metabolism	map00480	5/190	0.03960	*CD224*; *GST*; *ggt*; *PGD*; *OPLAH*
Ubiquinone and other terpenoid-quinone biosynthesis	map00130	2/190	0.04361	*CoQ5*; *CoQ2*
Glycosphingolipid biosynthesis-globo and isoglobo series	map00603	2/190	0.04361	*galA*; *HEXA_B*

### Identification of differentially expressed proteins (DEPs) using iTRAQ

The iTRAQ is a technique in quantitative proteome that accurately measures large-fold changes in protein expression within dynamic ranges of protein abundance (Juan et al. 2010). Here we used this technique to perform proteomic analyses of the key enzymes involved in AQ biosynthetic pathways. The iTRAQ data show that 44,751 unique peptides, which were assembled into 3987 proteins (Additional file 1: Figure S4). All proteins were then assigned into three main GO categories: biological process (BP), cellular component (CC), and molecular function (MF). As shown in Additional file 1: Figure S5, a large number of proteins were assigned into the GO categories of biological process and cellular component. Particularly, the proteins of more than 1000 involved in metabolic process, cellular process, single-organism metabolic process, cell, cell part, organelle, catalytic activity, and binding. In total, 3914 proteins did not change significantly, 24 proteins were downregulated, and 49 proteins were upregulated in FM10 compared to KB10 (Additional file 1: Figure S6).

**Fig. 4:**
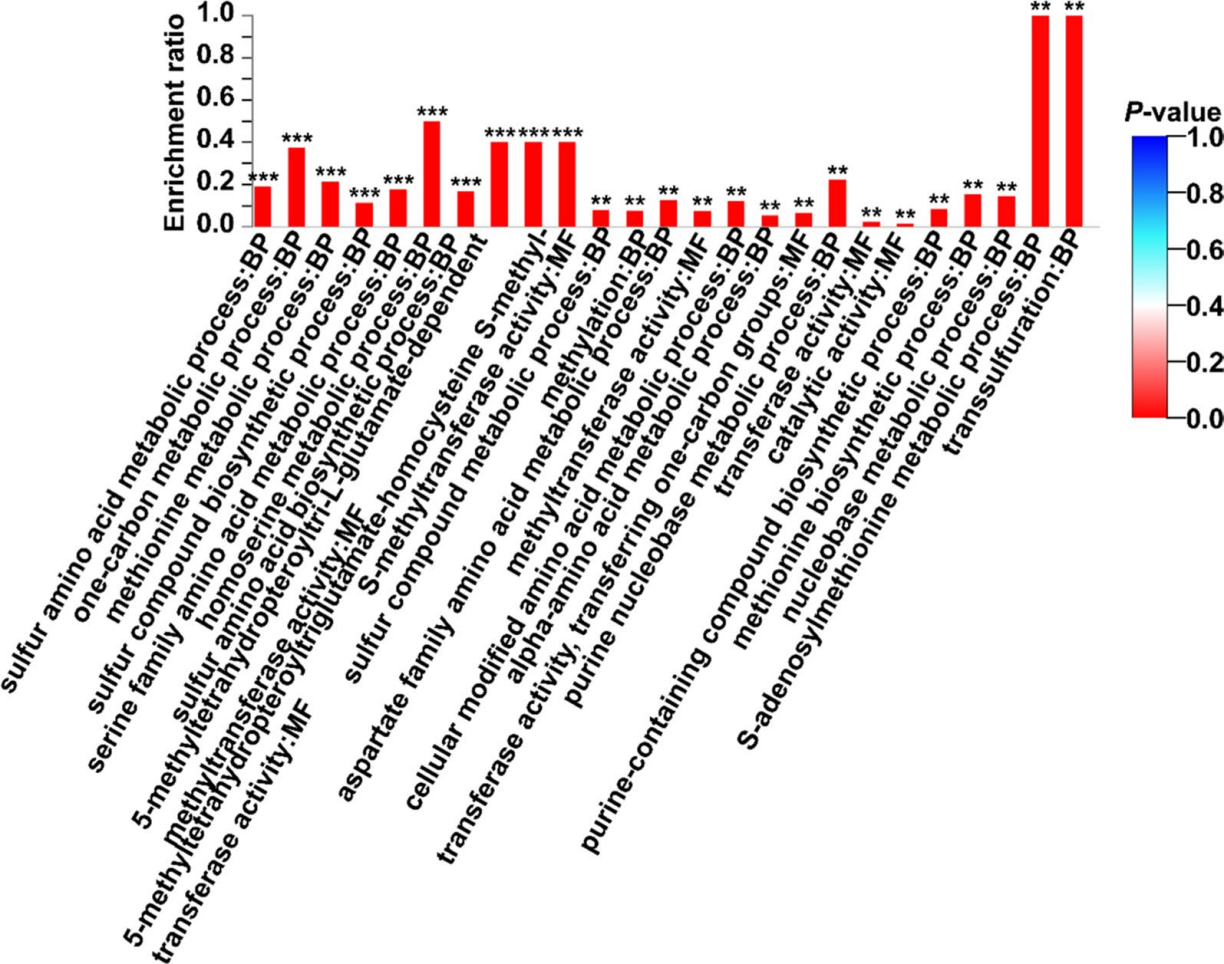
GO annotation of upregulated proteins on day 10. *P < 0.05, **P < 0.01

GO analysis was also used to further elucidate the putative functions of upregulated DEPs on day 10 (Fig. 4). According to the GO analysis, the upregulated DEPs in FM were mainly grouped into the following functions (number of proteins): sulfur amino acid metabolic process (4), one-carbon metabolic process (3), methionine metabolic process (3), sulfur compound biosynthetic process (4), serine family amino acid metabolic process (3), homoserine metabolic process (2), sulfur amino acid biosynthetic process (3), 5-methyltetrahydropteroyltri-l-glutamate-dependent methyltransferase activity (2), 5-methyltetrahydropteroyltriglutamate-homocysteine *S*-methyltransferase activity (2), and *S*-methyltransferase activity (2) (*P *< 0.001, Fig. 4 and Additional file 1: Table S3).

**Fig. 5:**
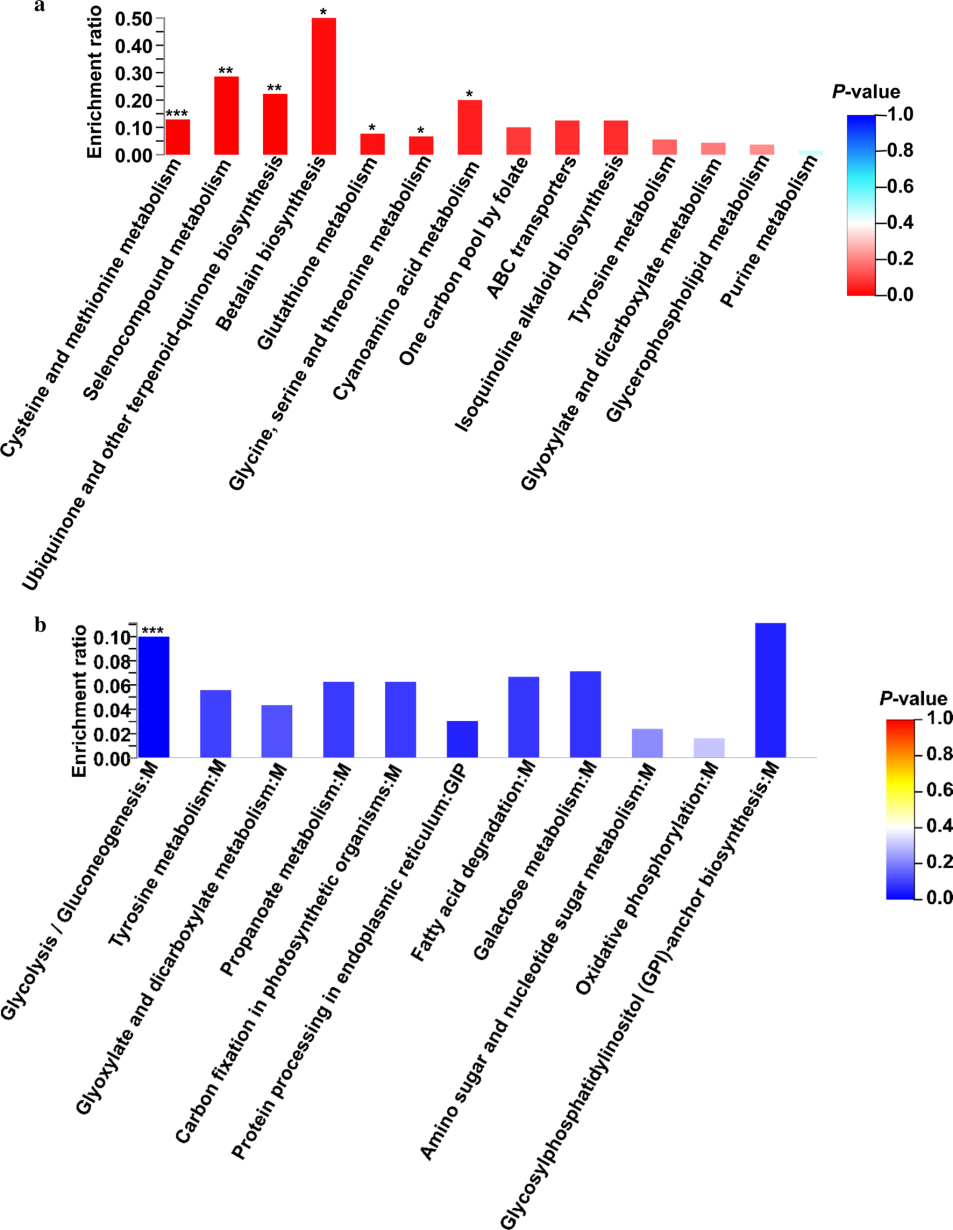
Enriched pathways of upregulated (**a**) and downregulated (**b**) proteins on day 10 based on KEGG annotation. *P < 0.05, **P < 0.01, ***P < 0.001

KEGG pathway enrichment analysis was also performed to investigate the enriched pathways of DEPs on day 10. Seven KEGG pathways were significantly upregulated in FM: cysteine and methionine metabolism (*P *< 0.001), selenocompound metabolism, ubiquinone and other terpenoid-quinone biosynthesis (*P* < 0.01), betalain biosynthesis, glutathione metabolism, glycine, serine and threonine metabolism, and cyanoamino acid metabolism (*P* < 0.05, Fig. 5a). One KEGG pathway was significantly downregulated: glycolysis/gluconeogenesis (*P* < 0.001, Fig. 5b).

Among these upregulated DEPs in FM, we further identified key enzymes that might be related to the biosynthesis of AQ (Table 2 and Additional file 1: Table S4). Five enzymes (ec:2.5.1.6, ec:2.5.1.18, ec:2.5.1.39, ec:2.5.1.21, and ec:2.5.1.54) are relate to transfer alkyl or aryl groups. Two enzymes (ec:2.1.1.14 and ec:2.1.1.41) are methyltransferases. Seventeen of them are oxidoreductases. The ec:2.1.2.1 and ec:2.1.2.10 are relate to hydroxymethyltransferase and aminomethyltransferase, respectively. Four enzymes (ec:2.3.3.1, ec:2.3.3.8, ec:2.3.3.13, and ec:6.2.1.1) can generate acetyl-CoA during the catalytic reaction.

**Table 2:** Critical upregulated proteins based on proteomic analysis (10-day samples)

Enzyme ID	Gene	*P*-value	Function	KEGG	Class
ec:2.5.1.6	*metK*	0.008	*S*-Adenosylmethionine synthetase	K00789	Transferring alkyl or aryl groups, other than methyl groups
ec:2.5.1.18	*GST*	1.5 × 10^5^	Glutathione *S*-transferase	K00799	
ec:2.5.1.39	*CoQ2*	0.038	4-Hydroxybenzoate polyprenyltransferase	K06125	
ec:2.5.1.21	*FDFT1*	0.024	Farnesyl-diphosphate farnesyltransferase	K00801	
ec:2.5.1.54	*aroF*	0.010	3-Deoxy-7-phosphoheptulonate synthase	K01626	
ec:2.1.1.14	*metE*	0.002	5-Methyltetrahydropteroyltriglutamate-homocysteine methyltransferase	K00549	Methyltransferases
ec:2.1.1.41	*SMT1*	0.043	Sterol 24-C-methyltransferase	K00559	
ec:2.1.2.1	*SHMT*	0.003	Glycine hydroxymethyltransferase	K00600	Hydroxymethyl-, formyl- and related transferases
ec:2.1.2.10	*AMT*	0.014	Aminomethyltransferase	K00605	
ec:2.3.3.1	*CS*	0.015	Citrate synthase	K01647	Acyltransferases; Acyl groups converted into alkyl groups on transfer
ec:2.3.3.8	*ACLY*	0.029	ATP citrate (pro-*S*)-lyase	K01648	
ec:2.3.3.13	*IMS*	0.029	2-Isopropylmalate synthase	K01649	
ec:6.2.1.1	*ACSS1_2*	0.036	Acetyl-CoA synthetase	K01895	Forming carbon–sulfur bonds

### Integrated analyses of transcriptomic and proteomic datasets of *A. camphorata* S-29

**Fig. 6:**
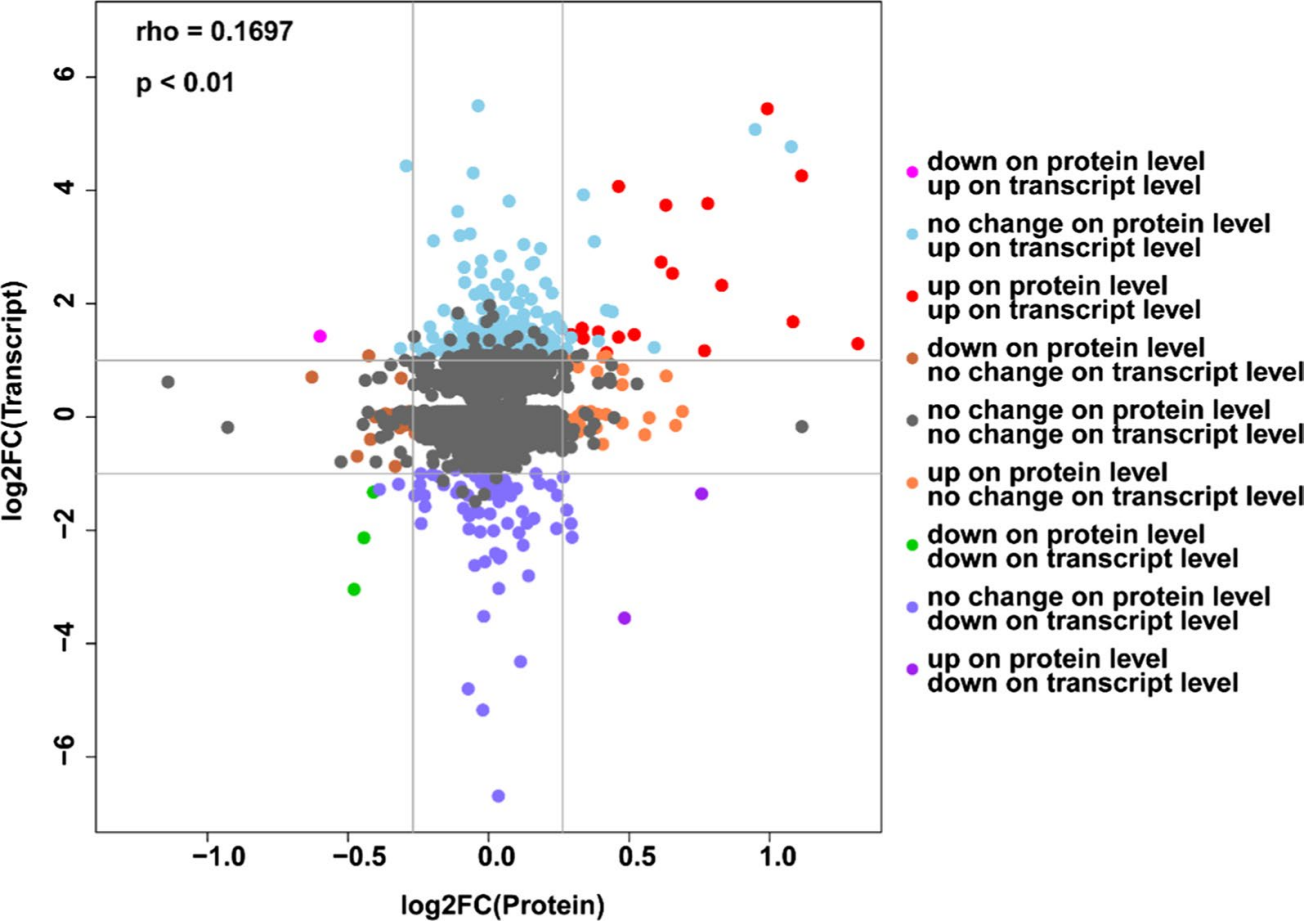
Correlation analysis of proteomic and transcriptomic data (10-day samples). Differentially expressed mRNAs or proteins are represented by solid dots

The correlation between the proteome and transcriptome of *A. camphorata* S-29 was demonstrated by scatter plot analysis (Fig. 6). The distribution of the dots with different colors shows that a large number (3589) of mRNAs and proteins did not exhibit changes in expression (black dots, Fig. 6 and Additional file 1: Table S5). On the other hand, a different trend was observed between protein and transcription levels. For instance, 253 genes were upregulated at the transcriptional level but unchanged at the protein level (sky blue). Moreover, 72 genes were downregulated at the transcriptional level but unchanged at the protein level (slate blue). Due to the regulations of transcription and translation, the mRNA expression levels are not always consistent with protein levels (Washburn et al. 2003). It is noteworthy that 18 unigenes were upregulated at the transcriptional level and the protein level (red).

For the comparative analysis of proteomic and transcriptomic data, we focused on the 9 unigenes that were both upregulated in transcriptomic and proteomic sequencing (Table 3). The *ubiA* and *CoQ2* genes simultaneously participate in the formation of 4-hydroxybenzoate polyprenyltransferase. Of note, among the genes for synthesized benzenoids, *PKS63787* gene was the DEG that exhibited the greatest upregulation. Ac-8 cytochrome P450 (CYP) monooxygenase synthesis-related gene *P450* and methyltransferase-related gene *metE* were significantly upregulated in the proteome and transcriptome data. *E1.14.13.1*, *E1.14.13.7*, and *ADK* were associated with the electron transport respiratory chain and energy metabolism.

**Table 3:** Significantly upregulated genes and proteins on day 10

Genes	Enzymes	Pro_log2FC	Pro_*P*-value	Rna_log2FC	Rna_*P*-value	Function
*ubiA*	ec:2.5.1.39	0.63	0.038	2.15	1.59E−48	4-Hydroxybenzoate polyprenyltransferase
*CoQ2*	ec:2.5.1.39	0.40	0.045	0.61	7.27E−07	4-Hydroxybenzoate polyprenyltransferase
*PKS63787*	PKS63787	0.44	0.036	3.51	9.49E−105	Synthesize several benzoquinones and benzenoids
*P450*	P450 monooxygenas	1.06	0.035	1.10	1.29E−16	ac-8 cytochrome P450 monooxygenas
*metE*	ec:2.1.1.14	0.32	0.002	0.92	1.84E−12	5-Methyltetrahydropteroyltriglutamate–homocysteine methyltransferase
*E1.14.13.1*	ec:1.14.13.1	0.76	0.027	3.47	2.73E−78	Salicylate hydroxylase
*E1.14.13.7*	ec: 1.14.13.7	0.61	0.002	2.65	2.09E−68	Phenol 2-monooxygenase (NADPH)
*ADK*	ec:2.7.1.20	0.27	0.014	0.75	9.61E−09	Adenosine kinase
*ahcY*	ec:3.3.1.1	0.37	0.010	0.98	9.87E−14	Adenosylhomocysteinase

*FC* fold change

### Validation of gene expression data using q-PCR

**Fig. 7:**
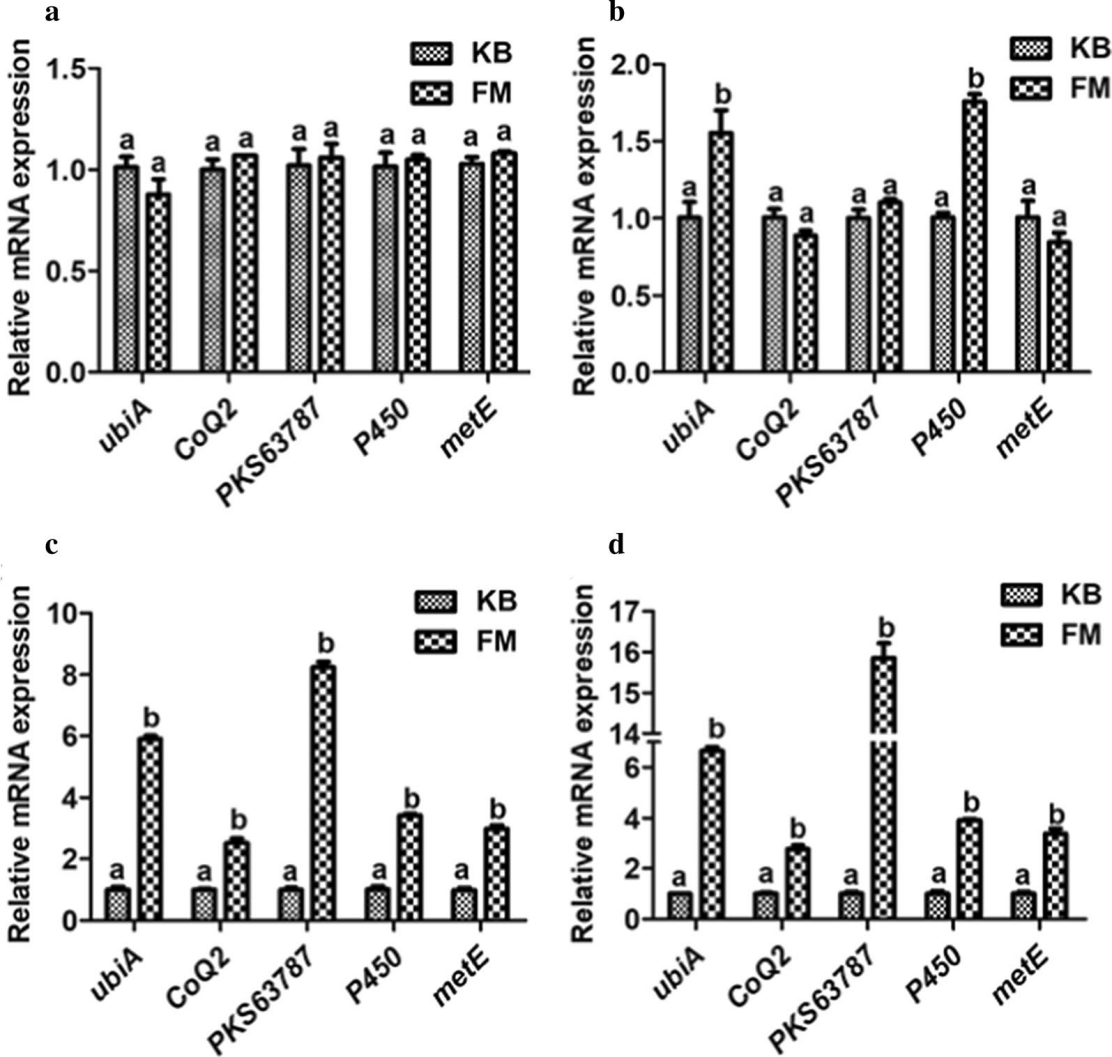
The q-PCR analysis of mRNA expression of genes potentially related to the biosynthesis of AQ on days 4 (**a**), 5 (**b**), 8 (**c**), and 10 (**d**). Means with different letters differed significantly (*P* < 0.05). The experiments were carried out with three biological repeats. Values are given as the means ± standard deviations (n = 3)

The mRNA expression levels of 5 genes in potential AQ biosynthesis on days 4, 5, 8, and 10 were studied (Fig. 7). The mRNA expression levels of 5 genes (*ubiA*, *CoQ2*, *PKS63787*, *P450*, and *metE*) in KB and FM were not significantly increased on day 4 (Fig. 7a, P > 0.05). Compared with the KB, the expression levels of the *ubiA* and *P450* genes increased significantly in FM on day 5 (Fig. 7b, *P* < 0.05). The expression levels of the *ubiA*, *CoQ2*, *PKS63787*, *P450*, and *metE* genes were significantly higher in FM than in KB at 8 and 10 days (Fig. 7c, d, *P* < 0.05). Therefore, the gene expression levels with the transcriptome sequencing analyses were consistent with those of the q-PCR analyses (Table 3 and Additional file 1: Table S6). The production of AQ increased significantly on 5–10 days compared with 4 and 5 days (Additional file 1: Figure S7). The AQ synthesis-related genes expression levels are in line with its production.

## Discussion

AQ is considered one of the most bioactive metabolites in *A. camphorata* (Lu et al. 2014). The addition of the AQ precursor (coenzyme Q_0_, orsellinic acid, etc.) via submerged fermentation is considered the most efficient method for the industrial production of AQ. In this study, we used transcriptomic and proteomic data to reveal AQ synthesis-related genes and enzymes during FM. GO enrichment and KEGG pathway analyses of transcriptomic data provided insights into the secondary metabolite biosynthesis pathways. The GO assignment system was used to obtain functional information for the DEGs, further assisting in understanding the distribution of DEG functions at a macro level. Given that fewer differential genes were noted between KB and FM groups in the early stage of adding coenzyme Q_0_, the number of genes on days 4 and 5 annotated in the GO categories were significantly less than that on day 10 (Figs. 1 and 2). Numerous genes involved in metabolic processes were significantly enriched in FM compared to KB on day 10. This finding indicates that the addition of the precursor coenzyme Q_0_ significantly increased the synthesis of metabolites.

KEGG pathway analysis provides classifications that are valuable for studying the complex biological functions of genes. KEGG analysis of transcriptomic data showed that the pathway of fatty acid degradation was significantly enriched in FM (Fig. 3). The *ECHS1* gene, which encodes enoyl-CoA hydratase, and the *atoB* gene, which encodes acetyl-CoA C-acetyltransferase, were upregulated. In fungi, the key metabolic pathway for fatty acid degradation is beta-oxidation. Fatty acids are broken down in a repeating process involving four steps in mitochondria, and each round of processing removes two carbons, in the form of acetyl-CoA, from the fatty acid chain (Shen and Burger 2009). In addition, pyruvate is oxidized to acetyl-CoA in mitochondria during pyruvate metabolism. *ACACA*, *E2.3.3.10*, *OXCT*, *IVD*, and *PAAH* genes, which are associated with acetyl-CoA, were also upregulated. The formation of AQ may be closely related to the ubiquinone biosynthesis pathway (Hu et al. 2016). The initial biosynthetic pathway of ubiquinone involves the conversion of three acetyl-coA units into farnesyl diphosphate (FPP) (Ericsson et al. 1992). Therefore, acetyl-CoA may enter either the mevalonate pathway or polyketide pathway, which form the farnesyl tail precursor or ring precursor of AQ in *A. camphorata* S-29, respectively (Chou et al. 2017). The *CoQ2* and *ubiA* genes encode *p*-hydroxybenzoate polyprenyl transferase, which is required in the biosynthetic pathway of ubiquinone (Forsgren et al. 2004; Liu et al. 2017). *P*-hydroxybenzoate polyprenyl transferase, which is also referred to as the ‘*CoQ2* enzyme’, mediates the second step in the final reaction sequence of coenzyme Q biosynthesis, namely, the condensation of the polyisoprenoid side chain with 4-*p*-hydroxybenzoate (Forsgren et al. 2004). The coupling of the aromatic substrate and isoprenoid chain is presumed to be the rate-limiting step in ubiquinone synthesis. Chou et al. (2019) proposed that the farnesylation of coenzyme Q_0_ at C-3 forms 5-demethoxy-coenzyme Q_3_ or coenzyme Q_3_, which is further reduced to form AQ. Therefore, we hypothesize that overexpression of *CoQ2* and *ubiA* genes promoted the linkage of coenzyme Q_0_ and the polyisoprene side chain, thus increasing AQ synthesis during FM.

The iTRAQ has the advantages of high coverage, accuracy, and sensitivity. Proteomic analyses of *A. camphorata* S-29 using the iTRAQ technique could provide useful information for understanding the AQ biosynthesis pathway. Based on the numerous unique proteins identified in GO functional categories, the largest categories for the functional group were sulphur amino acid and sulphur compound biosynthetic processes (Fig. 4). In microorganisms, sulphate is used in the synthesis of organic sulphur metabolites, mostly cysteine, methionine, and *S*-adenosylmethionine. The sulphur amino acids (SAA) include methionine and cysteine (Glenda et al. 2012). Methionine is metabolized via three major metabolic pathways: transmethylation, remethylation, and transsulfuration (Vigneaud et al. 1994). Fungi utilize the methyl group of methionine for biological methylation. Methionine serves as the major methyl group donor. The expression of *S*-adenosylmethionine synthetase is significantly increased during FM of *A. camphorata* S-29 compared with control (Fig. 4). *S*-adenosylmethionine synthetase is the transferring enzyme with *S*-methyltransferase activity. In addition, upregulated *metE* genes could encode an enzyme (ec:2.1.1.14) with methyltransferase activity. According to the reported results, it is important to have a sufficient supply of methyl groups for AQ structure formation (Wijayasinghe et al. 2014; Hu et al. 2016). These results suggest that *S*-adenosylmethionine synthetase may play a vital role in the biosynthesis of AQ.

According to results from Yu et al. (2016), the biosynthesis of aromatic compounds in *A. camphorata* occurs via the polyketide pathway. *PKS63787* plays a critical role in the biosynthesis of benzoquinones and benzenoids. Three metabolites, including benzenoids, benzoquinones, and AQ, are related to *PKS63787*. Yu et al. (2017) used phylogenetic analysis to demonstrate that *PKS63787* likely encodes an orsellinic acid synthase. The biosynthetic route of AQ follows a synthetic sequence similar to that of yeast coenzyme Q_6_. The pathway starts with the farnesylation of orsellinic acid to form 3-farnesyl-orsellinic acid, which undergoes further ring modification to form 5-demethoxy-coenzyme Q_3_, coenzyme Q_3_, and finally AQ (Chou et al. 2017). Table 3 and Additional file 1: Table S2 show that *PKS63787* gene expression is significantly increased in FM on day 10 (*P* < 0.001). After adding coenzyme Q_0_ on day 4, its content gradually decreased from days 4–10. Coenzyme Q_0_ was completely utilized after day 10 (Additional file 1: Figure S7). Therefore, we hypothesized that excess polyisoprene side chain induced the overexpression of the *PKS63787* gene to synthesize the cyclohexanone moiety of AQ. However, further investigations are required.

CYP genes are ubiquitously distributed genes in the genomes of almost all organisms. CYP genes encode a superfamily of haeme-containing monooxygenases that metabolize a wide variety of endogenous and xenobiotic compounds (Bernhardt 2006). Many fungal species have evolved complex and diverse CYP systems that play an important role in primary and secondary metabolism, allowing these fungi to exhibit unique and superior metabolic functions (Brink et al. 1998). CYP enzyme systems of fungi also include a wide range of catalysed reactions, such as reduction, heteroatom oxygenation, epoxidation, dealkylation, aromatic hydroxylation, and carbon hydroxylation (Brink et al. 1998; Ling et al. 2017). Hsu et al. (2011) identified 10 CYP genes (ac-1 to ac-10) from *A. camphorata*. According to phylogenetic tree of fungal CYP amino acid sequences, ac-8 CYP was assigned to a new CYP family. Since ac-8 CYP did not exhibit close evolutionary relationships with known CYPs, the function of ac-8 CYP is unknown. In the transcriptomic and proteomic analysis, the expression of ac-8 CYP monooxygenase increased significantly on day 10. In addition, mRNA expression levels of *P450* were significantly increased in the FM compared to the KB at 5, 8 and 10 days (Fig. 6b–d). Therefore, we hypothesized that ac-8 CYP monooxygenase plays an important role in AQ synthesis.

According to several reports, coenzyme Q_0_ is associated with the electron transport respiratory chain (Redfeam and Burgos 1966). Several DEGs and DEPs that might be closely related to the electron transport respiratory chain were noted in this study. *E1.14.13.1*, *E1.14.13.7*, and *ADK* are three energy-related genes that exhibit increasing levels of expression upon the addition of coenzyme Q_0_. One of the main functions of these genes is to provide energy for cells and electron transport. Farnesylation is a chemical reaction that involves covalent bonding, requiring higher energy. Overexpression of these genes may facilitate oxidative phosphorylation and accelerate electron transport through cytochrome c within the inner mitochondrial membrane, and finally activate ATP synthase to synthesise the energy carrier ATP. In addition to farnesylation steps, the conversion step of coenzyme Q_3_ to AQ involves the elimination of one double bond and the reduction of a keto group. These chemical reactions require several protons that are derived from NADH. When NADH is transformed into NAD+, this reaction releases two electrons, which can be accepted by electrophile substances, such as oxygen (Wei et al. 2020). This reaction may be beneficial for the conversion of coenzyme Q_3_ to AQ.

In summary, transcriptomic and proteomic analyses showed that specific genes and enzymes associated with ubiquinone and other terpenoid-quinone biosynthesis pathways as well as various transcription factors are involved in AQ formation. Two “omics” levels of analyses identified several important candidate genes and enzymes that regulate or participate in AQ biosynthesis (Additional file 1: Figure S8). This study is beneficial to analyse AQ biosynthesis pathways and provide strategies for targeted increases in AQ production by genetic means in the future.

## Supplementary information

**Additional file 1: Table S1.** Primers used for q-PCR. **Table S2.** Summary of DEGs in KEGG pathway annotation (10-day samples). **Table S3.** GO annotation of upregulated proteins (10-day samples). **Table S4.** Other upregulated proteins on day 10 based on proteomic analysis. **Table S5.** The number of genes and proteins at transcriptomic and proteomic levels (10-day samples). **Table S6.** The mRNA expression levels of genes on days 4 and 5. **Figure S1.** Venn diagram of expressed genes in *A. camphorata* S-29 transcriptomes in KB and FM on days 4, 5, and 10, respectively. **Figure S2.** Functional categorization by KEGG of *A. camphorata* S-29 transcriptome. **Figure S3.** Analysis of DEGs between KB and FM by KEGG enrichment map. A. KB4 VS FM4; B. KB5 VS FM5. **Figure S4.** A summary of protein information of *A. camphorata* S-29. **Figure S5.** The GO annotation of 3987 proteins in *A. camphorata* S-29. **Figure S6.** Volcano plot of the 3987 proteins during FM compared with KB. **Figure S7.** The concentrations of CoQ0 and AQ. The experiments were carried out with three replications. Values are given as the means ± standard deviations (n = 3). **Figure S8.** Genes annotations in the AQ synthesis pathway during FM. FPP, farnesyl diphosphate; OA, orsellinic acid; FOA, 3-farnesyl-orsellinic acid; 5-DMQ3, 5-demethoxy- coenzyme Q3; CoQ3, coenzyme Q3; AQ, antroquinonol.

## Data Availability

The FASTQ format raw reads were deposited to the National Center for Biotechnology Information Short Read Archive (NCBI SRA) database (accession: PRJNA543624 and PRJNA622907). The mass spectrometry proteomics data have been deposited to the ProteomeXchange Consortium (http://proteomecentral.proteomexchange.org) via the iProX partner repository with the dataset identifier PXD018004.
