# Supplementary material for: Comprehensive transcriptomic and proteomic analyses of antroquinonol biosynthetic genes and enzymes in Antrodia camphorata
Source: AMB Express. 2020 Aug 3;10:136. doi: 10.1186/s13568-020-01076-6 (PMC7399014; doi:10.1186/s13568-020-01076-6)
Supplement: Supplementary file 1 — Additional file 1: Table S1. Primers used for q-PCR. Table S2. Summary of DEGs in KEGG pathway annotation (10-day samples). Table S3. GO annotation of upregulated proteins (10-day samples). Table S4. Other upregulated proteins on day 10 based on proteomic analysis. Table S5. The number of genes and proteins at transcriptomic and proteomic levels (10-day samples). Table S6. The mRNA expression levels of genes on days 4 and 5. Figure S1. Venn diagram of expressed genes in A. camphorata S-29 transcriptomes in KB and FM on days 4, 5, and 10, respectively. Figure S2. Functional categorization by KEGG of A. camphorata S-29 transcriptome. Figure S3. Analysis of DEGs between KB and FM by KEGG enrichment map. A. KB4 VS FM4; B. KB5 VS FM5. Figure S4. A summary of protein information of A. camphorata S-29. Figure S5. The GO annotation of 3987 proteins in A. camphorata S-29. Figure S6. Volcano plot of the 3987 proteins during FM compared with KB. Figure S7. The concentrations of CoQ0 and AQ. The experiments were carried out with three replications. Values are given as the means ± standard deviations (n = 3). Figure S8. Genes annotations in the AQ synthesis pathway during FM. FPP, farnesyl diphosphate; OA, orsellinic acid; FOA, 3-farnesyl-orsellinic acid; 5-DMQ3, 5-demethoxy- coenzyme Q3; CoQ3, coenzyme Q3; AQ, antroquinonol. [file 13568_2020_1076_MOESM1_ESM.pdf]

AMB Express

Comprehensive transcriptomic and proteomic analyses of antroquinonol biosynthetic genes and enzymes in *Antrodia camphorata*

Xiaofeng Liu, Yongjun Xia, Yao Zhang, Caiyun Yang, Zhiqiang Xiong, Xin Song, Lianzhong Ai *

Shanghai Engineering Research Center of Food Microbiology, School of Medical Instrument and Food Engineering, University of Shanghai for Science and Technology, Shanghai 200093, PR China.

*Corresponding to: Professor Lianzhong Ai,

Address: University of Shanghai for Science and Technology, 516 Jungong Road, Shanghai 200093, China

E-mail address: ailianzhong1@126.com

Tel: +86-21-55897302

Fax: +86-21-55897302

ORCID: 0000-0002-6681-9102

Table S1 Primers used for q-PCR.

Primer name	Sequence (5' → 3')	Product length (bp)	Tm (°C)
18S rRNA-F	AACTTGTCGAATCGCATGGC	119	55
18S rRNA-R	ACCCTTATTCCCCGTTACCC		
ubiA-F	GCAGTGATATACCTCGTCGCTT	121	55
ubiA-R	CGGATGCTATGGGTCGATTC		
COQ2-F	TGGCCCTGACATGGTCTTCT	123	55
COQ2-R	CCGATTGTGCAGCCGATGAA		
PKS63787-F	GTCAAAGCTCACGTCATGGAGG	122	55
PKS63787-R	CCCTTGTGCGTTGCGTCGATA		
P450-F	GAGAAACTATTCGTCTCGCAGGT	124	55
P450-R	CATCTGGAGTATCGCACCAC		
metE-F	AGCGGCGACTTCTCCCTATATG	121	55
metE-R	CACGGCCCATGGCGAAATAAAC		

Table S2 Summary of DEGs in KEGG pathway annotation (10-day samples).

Gene name	KB10 fpkm	FM10 fpkm	*P*-value	FDR	regulate	GOs	COG	KOG	KO/Gene ID	Definition
lldD	18.47	28.58	1.27E-05	1.22E-04	up	GO:0046872;GO:0020037;GO:0010181;GO:0055114;GO:0016491	COG5274;COG1304	KOG0537;KOG0538	K00101	L-lactate dehydrogenase (cytochrome)
IMS	716.99	1487.54	1.55E-15	6.16E-14	up	GO:0009099;GO:0009098;GO:0006090;GO:0003852;GO:0009097	COG0119	KOG2367	K01649	2-isopropylmalate synthase
ACACA	34.64	59.14	1.89E-09	3.80E-08	up	GO:0003989;GO:0004075;GO:0046872;GO:0006090;GO:0005524;GO:0006633;GO:0009343	COG0439;COG4799;COG0511	KOG0368	K11262	acetyl-CoA carboxylase / biotin carboxylase 1
atoB	487.05	805.89	7.14E-09	1.35E-07	up	GO:0055085;GO:0016021	COG0477;COG0183	KOG2533;KOG1390	K00626	acetyl-CoA C-acetyltransferase
FH	168.38	246.20	7.95E-06	8.03E-05	up	GO:0019643;GO:0045239;GO:0004333;GO:0006106	COG0114	KOG1317	K01679	fumarate hydratase, class II
ALDH	595.25	3113.98	4.29E-60	1.38E-57	up	GO:0006561;GO:0006525;GO:0016620;GO:0055114;GO:0003842	COG1012	KOG2450	K00128	aldehyde dehydrogenase (NAD+)
maeB	149.94	235.46	2.63E-07	3.67E-06	up	GO:0006099;GO:0004471;GO:0015976;GO:0046872;GO:0006090;GO:0051287;GO:0006108	COG0281	KOG1257	K00029	malate dehydrogenase (oxaloacetate-decarboxylating)(NADP+)
pckA	452.35	672.51	2.45E-06	2.82E-05	up	GO:0006099;GO:0015976;GO:0016310;GO:0005524;GO:0016301;GO:0006094;GO:0004612	COG1866	_	K01610	phosphoenolpyruvate carboxykinase (ATP)
amiE	19.10	38.57	2.66E-11	6.77E-10	up	GO:0008152;GO:0016884	COG0154	KOG1212;KOG1211	K01426	amidase
PRODH	59.71	71.12	1.29E-02	4.08E-02	up	GO:0004553;GO:0044710;GO:0005976	COG0506	KOG0186	K00318	proline dehydrogenase
speB	112.73	179.48	2.44E-07	3.43E-06	up	GO:0006807;GO:0046872;GO:0016813	COG0010	KOG2964	K01480	agmatinase
TPS	30.08	59.05	7.57E-13	2.34E-11	up	GO:0005992;GO:0016740	COG0380;COG1877	KOG1050	K16055	trehalose 6-phosphate synthase/phosphatase
GPI	217.62	619.37	2.02E-27	1.67E-25	up	GO:0004347;GO:0006098;GO:0005982;GO:0006094;GO:0006096;GO:0005985	COG0166	KOG2446	K01810	glucose-6-phosphate isomerase
E3.2.1.58	29.98	81.94	3.24E-23	2.17E-21	up	GO:0016787	COG2730	_	K01210	glucan 1,3-beta-glucosidase
UGP2	12.84	13.06	7.78E-01	8.65E-01	up	GO:0008152;GO:0016779	_	_	K00963	UTP--glucose-1-phosphate uridylyltransferase
E2.4.1.34	79.30	112.79	2.15E-	1.91	up	GO:0005982;GO:0006508;	_	_	K00706	1,3-beta-glucan synthase

			05	E-04		GO:0006075;GO:0003843;GO:0005985;GO:0000148;GO:0004190				
AGL	23.02	36.81	1.45E-07	2.14E-06	up	GO:0004135;GO:0005980;GO:0005982;GO:0004134;GO:0005985	COG3408	KOG3625	K01196	glycogen debranching enzyme
bglX	16.71	29.41	1.23E-08	2.20E-07	up	GO:0004553;GO:0005975	COG1472	_	K05349	beta-glucosidase
SUV39H	3.17	4.38	1.87E-02	5.45E-02	up	_	_	_	K11419	[histone H3]-lysine9 N-trimethyltransferase SUV39H
OGDH	128.95	243.51	2.74E-12	7.97E-11	up	GO:0006099;GO:0045252;GO:0006568;GO:0006554;GO:0004591;GO:0030976	COG0567	KOG0450	K00164	2-oxoglutarate dehydrogenase E1 component
ECHS1	217.47	340.17	4.18E-07	5.64E-06	up	GO:0008152;GO:0003824	COG1024	KOG1680	K07511	enoyl-CoA hydratase
ggt	11.38	16.43	5.66E-04	3.10E-03	up	GO:0006691;GO:0019530;GO:0006749;GO:0006693;GO:0003840	COG0405	KOG2410	K00681	gamma-glutamyltranspeptidase / glutathione hydrolase
ACO	785.95	1358.02	5.72E-10	1.23E-08	up	GO:0003994;GO:0019643;GO:0051539;GO:0046487	COG1048	KOG0453	K01681	gamma-glutamyltranspeptidase / glutathione hydrolase
ACLY	136.90	411.29	4.20E-30	4.38E-28	up	GO:0004775;GO:0003878;GO:0005524;GO:0019643;GO:0042709;GO:0048037;GO:0044262	COG0074;COG0045;COG0372	KOG1254	K01648	ATP citrate (pro-S)-lyase
SDHA	79.52	131.18	1.20E-08	2.17E-07	up	GO:0016627;GO:0006099;GO:0022900;GO:0050660	COG1053	KOG2404;KOG2403	K00234	succinate dehydrogenase (ubiquinone) flavoprotein subunit
CS	382.07	552.91	8.81E-06	8.75E-05	up	GO:0006099;GO:0046487;GO:0044262;GO:0004108	COG0372	KOG2617	K01647	citrate synthase
E2.3.3.10	481.07	990.36	3.54E-15	1.39E-13	up	GO:0006574;GO:0008299;GO:0004421;GO:0006552;GO:0006084;GO:0046950;GO:0006550	COG3425	KOG1393	K01641	hydroxymethylglutaryl-CoA synthase
OXCT	95.40	147.41	4.84E-07	6.43E-06	up	GO:0006574;GO:0006552;GO:0005739;GO:0006550;GO:0008260;GO:0046952	COG1788;COG2057	KOG3822	K01027	3-oxoacid CoA-transferase
IVD	25.04	38.80	7.64E-05	5.70E-04	up	GO:0050660;GO:0006118;GO:0003995;GO:0055114	COG1960	KOG0141	K00253	isovaleryl-CoA dehydrogenase
HIBADH	28.65	54.79	1.28E-09	2.61E-08	up	GO:0004616;GO:0019521;GO:0008442;GO:0051287;GO:0006574;GO:0006098;GO:0006552;GO:0006550	COG2084	KOG0409	K00020	3-hydroxyisobutyrate dehydrogenase
XYLB	63.46	102.67	1.25E-07	1.87E-06	up	GO:0005975;GO:0016773	COG1070	KOG2531	K00854	xylulokinase

SORD	32.28	59.08	4.82E-09	9.27 E-08	up	GO:0008270;GO:0055114; GO:0016491	COG1063	KOG0024	K00008	L-iditol 2-dehydrogenase
ARD1	26.92	33.66	3.39E-02	8.82 E-02	up	GO:0008270;GO:0055114; GO:0016491	COG1063	KOG0024	K17818	D-arabinitol dehydrogenase (NADP+)
AOC3	64.04	125.68	4.82E-13	1.53 E-11	up	GO:0008131;GO:0005507; GO:0048038;GO:0009308; GO:0055114	COG3733	_	K00276	primary-amine oxidase
manC	106.67	100.11	8.81E-01	9.34 E-01	down	GO:0009058;GO:0016779	COG0836	_	K00971	mannose-1-phosphate guanylyltransferase
HEXA_B	76.02	119.83	4.73E-05	3.76 E-04	up	GO:0001575;GO:0004563; GO:0006040;GO:0005975; GO:0006027	_	_	K12373	hexosaminidase
E3.2.1.14	86.44	368.66	6.47E-48	1.24 E-45	up	GO:0006032;GO:0008061; GO:0016998;GO:0004568; GO:0005975	_	_	K01183	chitinase
E1.6.2.2	195.56	326.80	1.13E-08	2.06 E-07	up	GO:0055114;GO:0016491	COG0543	KOG0534	K00326	cytochrome-b5 reductase
CHS1	75.83	125.76	1.45E-08	2.57 E-07	up	GO:0006031;GO:0004100 GO:0020037;GO:0003677; GO:0008152;GO:0005524; GO:0003774;GO:0016459; GO:0016758	COG1215	KOG2571	K00698	chitin synthase
nagZ	16.02	23.72	2.17E-04	1.37 E-03	up	GO:0004553;GO:0005975	COG1472	_	K01207	beta-N-acetylhexosaminidase
abfA	1.56	2.70	6.16E-03	2.25 E-02	up	GO:0046556;GO:0046373; GO:0009117	COG3534	_	K01209	alpha-L-arabinofuranosidase
PAAH	28.02	38.24	1.58E-03	7.25 E-03	up	GO:0055114;GO:0018874; GO:0006574;GO:0070403; GO:0006552;GO:0006554; GO:0006568;GO:0003857; GO:0006633;GO:0006550	COG1250	KOG2304	K00074	3-hydroxybutyryl-CoA dehydrogenase
NIT-6	61.83	185.54	6.37E-28	5.45 E-26	up	GO:0042128;GO:0051537; GO:0020037;GO:0008942; GO:0050660;GO:0055114	COG1251	KOG1336	K17877	nitrite reductase (NAD(P)H)
NR	10.09	26.42	1.66E-20	9.23 E-19	up	GO:0097159;GO:1901363; GO:0046914;GO:0044710; GO:0050662;GO:0016491	COG2041	KOG0535	K10534	nitrate reductase (NAD(P)H)
FAS2	34.76	49.19	7.51E-04	3.90 E-03	up	GO:0000287;GO:0015940; GO:0006730;GO:0042967; GO:0055114;GO:0005835; GO:0004318;GO:0004089; GO:0008270;GO:0006633; GO:0008897	_	_	K00667	fatty acid synthase subunit alpha, fungi type
fabG	160.41	359.52	6.51E-	2.94	up	GO:0008152	COG1028	KOG0725	K00059	3-oxoacyl-[acyl-carrier protein]

			17	E-15						reductase
FAS1	25.71	29.15	4.85E-02	1.15E-01	up	GO:0000287;GO:0015940;GO:0006730;GO:0042967;GO:0055114;GO:0005835;GO:0004318;GO:0004089;GO:0008270;GO:0006633;GO:0008897	COG4981;COG0331	_	K00668	fatty acid synthase subunit beta, fungi type
GST	1.84	6.61	1.94E-05	1.76E-04	up	GO:0008152;GO:0016740	COG0625	KOG0406	K00799	glutathione S-transferase
PGD	1794.60	3637.97	9.51E-15	3.65E-13	up	GO:0050661;GO:0006098;GO:0004616;GO:0019521	COG0362	KOG2653	K00033	6-phosphogluconate dehydrogenase
OPLAH	40.65	71.16	3.59E-09	7.04E-08	up	GO:0008152;GO:0003824	COG0146	KOG1939	K01469	5-oxoprolinase (ATP-hydrolysing)
COQ5	172.91	114.09	1.83E-03	8.18E-03	down	GO:0008168;GO:0032259	COG2226	KOG1540	K06127	2-methoxy-6-polyprenyl-1,4-benzoquinol methylase
COQ2	2185.67	3333.84	7.27E-07	9.27E-06	up	GO:0047293 GO:0002083	COG0382	_	K06125	4-hydroxybenzoate polyprenyltransferase
galA	6.52	10.22	5.18E-04	2.88E-03	up	GO:0004553;GO:0005975;GO:0046486;GO:0006012;GO:0004557	_	KOG2366	K07407	alpha-galactosidase
pks63787	7.572	87.218	9.49E-105	7.48E-102	up	GO:0008152;GO:0003824	COG3321	KOG1394	-	synthesize several benzenoids

Table S3 GO annotation of upregulated proteins (10-day samples).

GO Description	Proteins Number	GO ID	GO Term	Ratio in study	*P*-value
sulfur amino acid metabolic process	4	GO:0000096	BP	4/29	4.12E-05
one-carbon metabolic process	3	GO:0006730	BP	3/29	4.72E-05
methionine metabolic process	3	GO:0006555	BP	3/29	0.000295
sulfur compound biosynthetic process	4	GO:0044272	BP	4/29	0.000328
serine family amino acid metabolic process	3	GO:0009069	BP	3/29	0.00054
homoserine metabolic process	2	GO:0009092	BP	2/29	0.00056
sulfur amino acid biosynthetic process	3	GO:0000097	BP	3/29	0.000643
5-methyltetrahydropteroyltri-L-glutamate-dependent methyltransferase activity	2	GO:0042085	MF	2/29	0.000928
5-methyltetrahydropteroyltriglutamate-homocysteine S-methyltransferase activity	2	GO:0003871	MF	2/29	0.000928
S-methyltransferase activity	2	GO:0008172	MF	2/29	0.000928
sulfur compound metabolic process	4	GO:0006790	BP	4/29	0.0013
methylation	4	GO:0032259	BP	4/29	0.001508
aspartate family amino acid metabolic process	3	GO:0009066	BP	3/29	0.001534
methyltransferase activity	4	GO:0008168	MF	4/29	0.00162
cellular modified amino acid metabolic process	3	GO:0006575	BP	3/29	0.001731
alpha-amino acid metabolic process	5	GO:1901605	BP	5/29	0.001786
transferase activity, transferring one-carbon groups	4	GO:0016741	MF	4/29	0.002416
purine nucleobase metabolic process	2	GO:0006144	BP	2/29	0.00326
transferase activity	11	GO:0016740	MF	11/29	0.004317
catalytic activity	23	GO:0003824	MF	23/29	0.004472
purine-containing compound biosynthetic process	3	GO:0072522	BP	3/29	0.004609
methionine biosynthetic process	2	GO:0009086	BP	2/29	0.006893
nucleobase metabolic process	2	GO:0009112	BP	2/29	0.007992
S-adenosylmethionine metabolic process	1	GO:0046500	BP	1/29	0.009894
transsulfuration	1	GO:0019346	BP	1/29	0.009894
cysteine biosynthetic process via cystathionine	1	GO:0019343	BP	1/29	0.009894
S-adenosylmethionine biosynthetic process	1	GO:0006556	BP	1/29	0.009894
L-methionine biosynthetic process from homoserine via O-acetyl-L-homoserine and cystathionine	1	GO:0019280	BP	1/29	0.009894
L-methionine biosynthetic process from L-homoserine via cystathionine	1	GO:0019279	BP	1/29	0.009894
endoplasmic reticulum inheritance	1	GO:0048309	BP	1/29	0.009894
methionine adenosyltransferase activity	1	GO:0004478	MF	1/29	0.009894
hydrolase activity, acting on ether bonds	1	GO:0016801	MF	1/29	0.009894
trialkylsulfonium hydrolase activity	1	GO:0016802	MF	1/29	0.009894
cystathionine gamma-lyase activity	1	GO:0004123	MF	1/29	0.009894
adenosine kinase activity	1	GO:0004001	MF	1/29	0.009894
adenosylhomocysteinase activity	1	GO:0004013	MF	1/29	0.009894
cellular amino acid metabolic process	5	GO:0006520	BP	5/29	0.01363

purine-containing compound metabolic process	3	GO:0072521	BP	3/29	0.017811
aspartate family amino acid biosynthetic process	2	GO:0009067	BP	2/29	0.01932
homocysteine metabolic process	1	GO:0050667	BP	1/29	0.019694
pore complex assembly	1	GO:0046931	BP	1/29	0.019694
'de novo' L-methionine biosynthetic process	1	GO:0071266	BP	1/29	0.019694
nuclear pore complex assembly	1	GO:0051292	BP	1/29	0.019694
purine ribonucleoside salvage	1	GO:0006166	BP	1/29	0.019694
endoplasmic reticulum tubular network organization	1	GO:0071786	BP	1/29	0.019694
glycine hydroxymethyltransferase activity	1	GO:0004372	MF	1/29	0.019694
nucleoside kinase activity	1	GO:0019206	MF	1/29	0.019694
purine ribonucleoside biosynthetic process	2	GO:0046129	BP	2/29	0.022804
purine nucleoside biosynthetic process	2	GO:0042451	BP	2/29	0.022804
transferase activity, transferring alkyl or aryl (other than methyl) groups	2	GO:0016765	MF	2/29	0.022804
alpha-amino acid biosynthetic process	3	GO:1901607	BP	3/29	0.023234
adenine biosynthetic process	1	GO:0046084	BP	1/29	0.0294
nucleoside salvage	1	GO:0043174	BP	1/29	0.0294
L-methionine biosynthetic process	1	GO:0071265	BP	1/29	0.0294
AMP biosynthetic process	1	GO:0006167	BP	1/29	0.0294
cortical endoplasmic reticulum	1	GO:0032541	CC	1/29	0.0294
glycosyl compound biosynthetic process	2	GO:1901659	BP	2/29	0.030481
nucleoside biosynthetic process	2	GO:0009163	BP	2/29	0.030481
ribonucleoside biosynthetic process	2	GO:0042455	BP	2/29	0.030481
cellular amino acid biosynthetic process	3	GO:0008652	BP	3/29	0.037755
adenine metabolic process	1	GO:0046083	BP	1/29	0.039013
tetrahydrofolate interconversion	1	GO:0035999	BP	1/29	0.039013
nuclear pore organization	1	GO:0006999	BP	1/29	0.039013
purine-containing compound salvage	1	GO:0043101	BP	1/29	0.039013
endoplasmic reticulum subcompartment	1	GO:0098827	CC	1/29	0.039013
endoplasmic reticulum tubular network	1	GO:0071782	CC	1/29	0.039013
tetrahydrofolate metabolic process	1	GO:0046653	BP	1/29	0.048534
cysteine biosynthetic process	1	GO:0019344	BP	1/29	0.048534
endoplasmic reticulum organization	1	GO:0007029	BP	1/29	0.048534
AMP metabolic process	1	GO:0046033	BP	1/29	0.048534
cysteine metabolic process	1	GO:0006534	BP	1/29	0.048534
carbohydrate transport	1	GO:0008643	BP	1/29	0.048534

BP, biological process; MF, molecular function; CC, cellular component. Ratio in study, the molecule is the number of DEPs enriched in the GO annotation, and the denominator is the total number of proteins enriched in the GO annotation by the upregulated protein set.

Table S4 Other upregulated proteins on day 10 based on proteomic analysis.

Enzyme ID	Gene	*P*-value	Function	KEGG	Class
ec:1.5.1.20	*metF*	0.02474	methylenetetrahydrofolate reductase (NADPH)	K00297	Oxidoreductases; With NAD+ or NADP+ as acceptor
ec:1.5.1.5	*MTHFD*	0.012	methylenetetrahydrofolate dehydrogenase (NADP+)	K00288	
ec:1.1.1.169	*panE*	0.0001	2-dehydropantoate 2-reductase	K00077	
ec:1.1.1.40	*maeB*	0.041	malate dehydrogenase (oxaloacetate-decarboxylating) (NADP+)	K00029	
ec:1.3.1.34	*DECR2*	0.041	peroxisomal 2,4-dienoyl-CoA reductase	K13237	
ec:1.3.1.22	*SRD5A1*	0.027	3-oxo-5-alpha-steroid 4-dehydrogenase 1	K12343	
ec:1.1.1.170	*NSDHL*	0.044	sterol-4alpha-carboxylate 3-dehydrogenase (decarboxylating)	K07748	
ec:1.7.1.4	*NIT-6*	0.023	nitrite reductase (NAD(P)H)	K17877	
ec:1.1.1.42	*IDH1*	0.020	isocitrate dehydrogenase	K00031	
ec:1.3.8.4	*IVD*	0.017	isovaleryl-CoA dehydrogenase	K00253	Oxidoreductases; With a flavin as acceptor
ec:1.3.8.7	*ACADM*	0.012	acyl-CoA dehydrogenase	K00249	
ec:1.4.4.2	*GLDC*	0.044	glycine dehydrogenase	K00281	Oxidoreductases; With a disulfide as acceptor
ec:1.14.14.92	*CYP53A1*	0.030	benzoate 4-monooxygenase	K07824	Oxidoreductases; With reduced flavin or flavoprotein as one donor, and incorporation of one atom of oxygen into the other donor
ec:1.14.13.1	*E1.14.13.1*	0.027	salicylate hydroxylase	K00480	Oxidoreductases; With NADH or NADPH as one donor, and incorporation of one atom of oxygen into the other donor
ec:1.14.13.7	*E1.14.13.7*	0.002	phenol 2-monooxygenase (NADPH)	K03380	
ec:1.14.18.9	*MESO1*	0.006	methylsterol monooxygenase	K07750	Oxidoreductases; With another compound as one donor, and incorporation of one atom of oxygen into the other donor
ec:1.14.18.1	*TYR*	0.044	tyrosinase	K00505	
ec:4.4.1.1	*CTH*	0.008	cystathionine gamma-lyase	K01758	Carbon-sulfur lyases
ec:2.2.1.6	*ilvB*	0.003	acetolactate synthase I/II/III large subunit	K01652	Transferring aldehyde or ketonic groups
ec:2.2.1.6	*ilvH*	0.027	acetolactate synthase I/III small subunit	K01653	

ec:2.7.1.20	*ADK*	0.014	adenosine kinase	K00856	Transferring phosphorus-containing groups
ec:2.7.11.11	*PKA*	0.020	protein kinase A	K04345	
ec:2.7.1.174	*DGK1*	0.013	diacylglycerol kinase (CTP)	K16368	
ec:2.7.11.1	*PDPK1*	0.031	3-phosphoinositide dependent protein kinase-1	K06276	
ec:4.1.1.49	*pckA*	0.024	phosphoenolpyruvate carboxykinase (ATP)	K01610	Carbon-carbon lyases
ec:3.3.1.1	*ahcY*	0.010	adenosylhomocysteinase	K01251	Thioether and trialkylsulfonium hydrolases

Table S5 The number of genes and proteins at transcriptomic and proteomic levels (10-day samples).

	Protein(down)	Protein(no change)	Protein(up)
Gene(up)	1	253	18
Gene(no change)	20	3589	29
Gene(down)	3	72	2

Table S6 The mRNA expression levels of genes on days 4 and 5.

Gene	log2FC(FM4/KB4)	*P*-value	significant	regulate	log2FC(FM5/KB5)	p-value	significant	regulate
ubiA	-0.44	0.05	no	down	0.68	0.001	no	up
COQ2	0.15	0.30	no	up	-0.46	0.001	no	down
pks63787	0.18	0.28	no	up	0.23	0.31	no	up
E1.14.13.1	-0.28	0.23	no	down	0.2	0.42	no	up
P450	0.06	0.63	no	up	0.56	0.0004	no	up
metE	0.15	0.25	no	up	-0.45	0.0009	no	down
E1.14.13.7	-0.09	0.67	no	down	0.07	0.84	no	up
ahcY	0.13	0.32	no	up	-0.37	0.006	no	down
ADK	0.1	0.45	no	up	-0.22	0.09	no	down

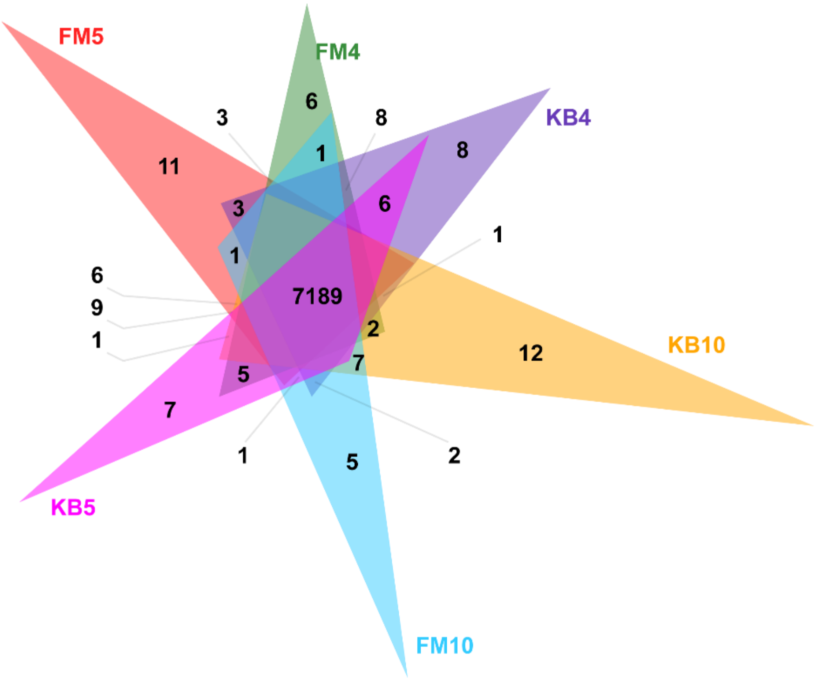

Fig. S1 Venn diagram of expressed genes in *A. camphorata* S-29 transcriptomes in KB and FM on days 4, 5, and 10, respectively.

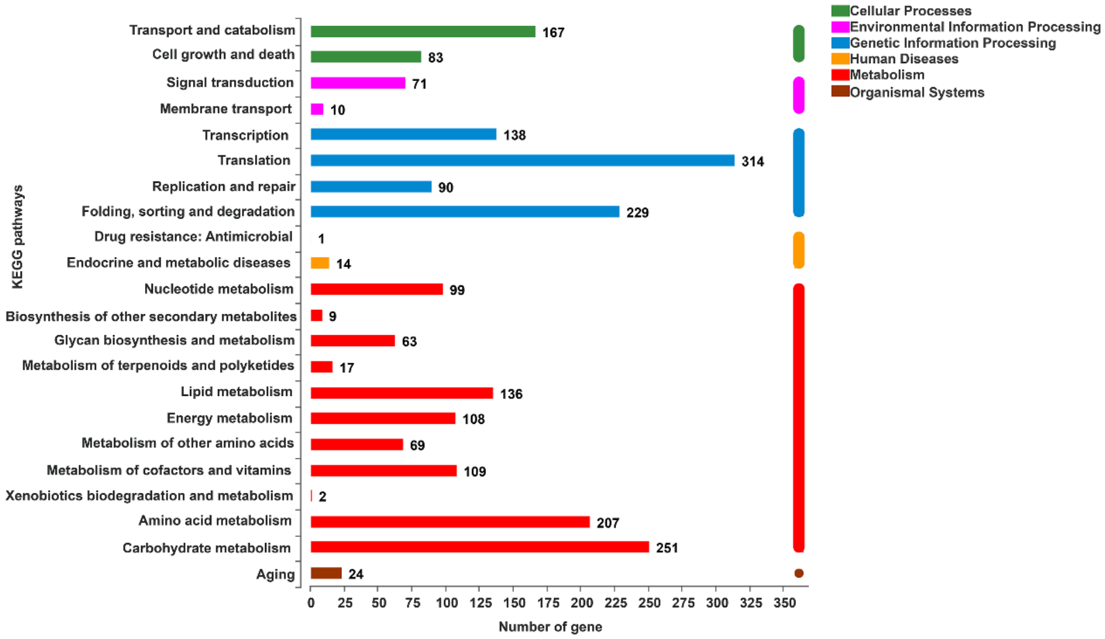

Fig. S2 Functional categorization by KEGG of *A. camphorata* S-29 transcriptome.

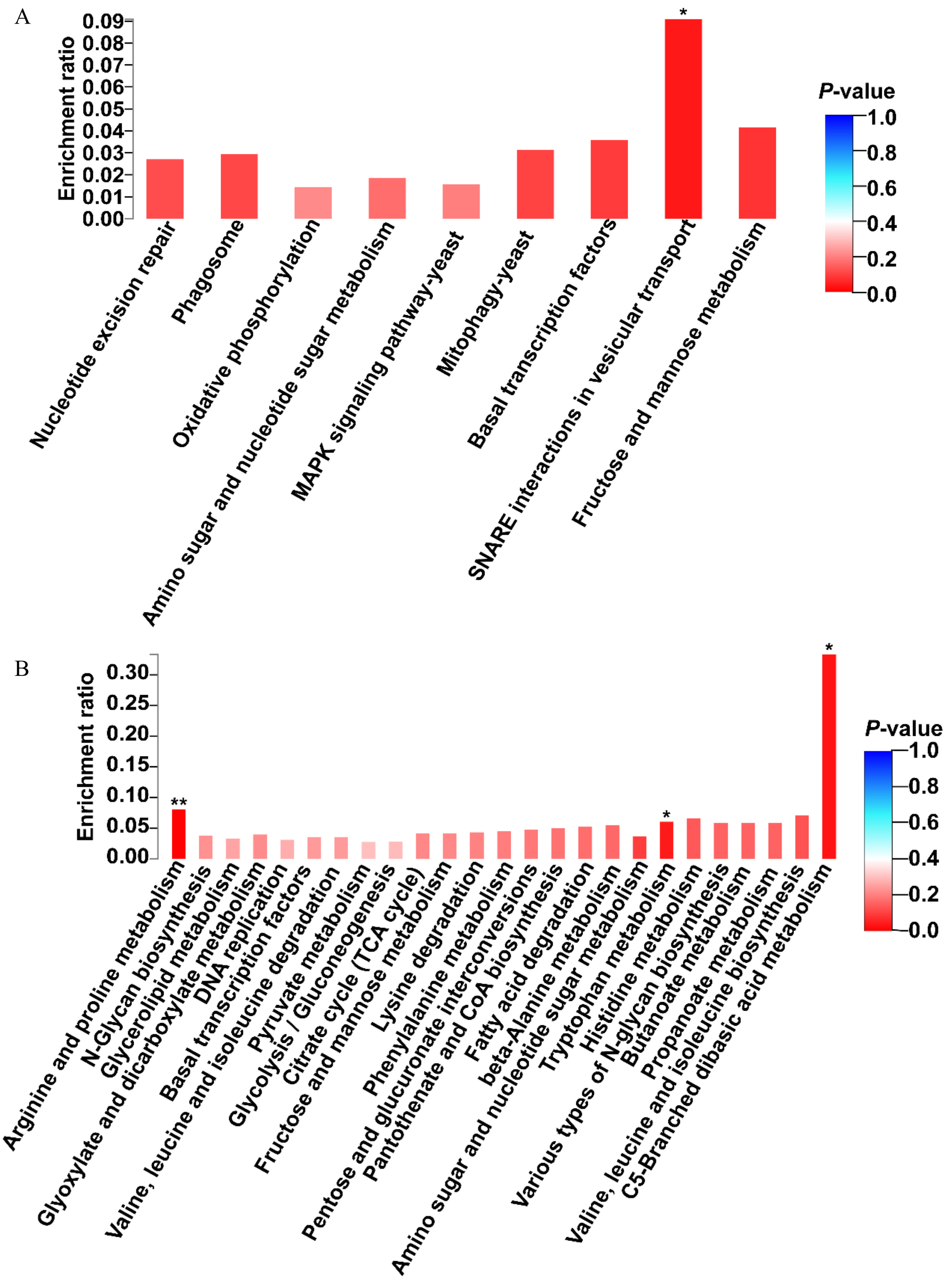

Fig. S3 Analysis of DEGs between KB and FM by KEGG enrichment map. A. KB4 VS FM4; B. KB5 VS FM5.

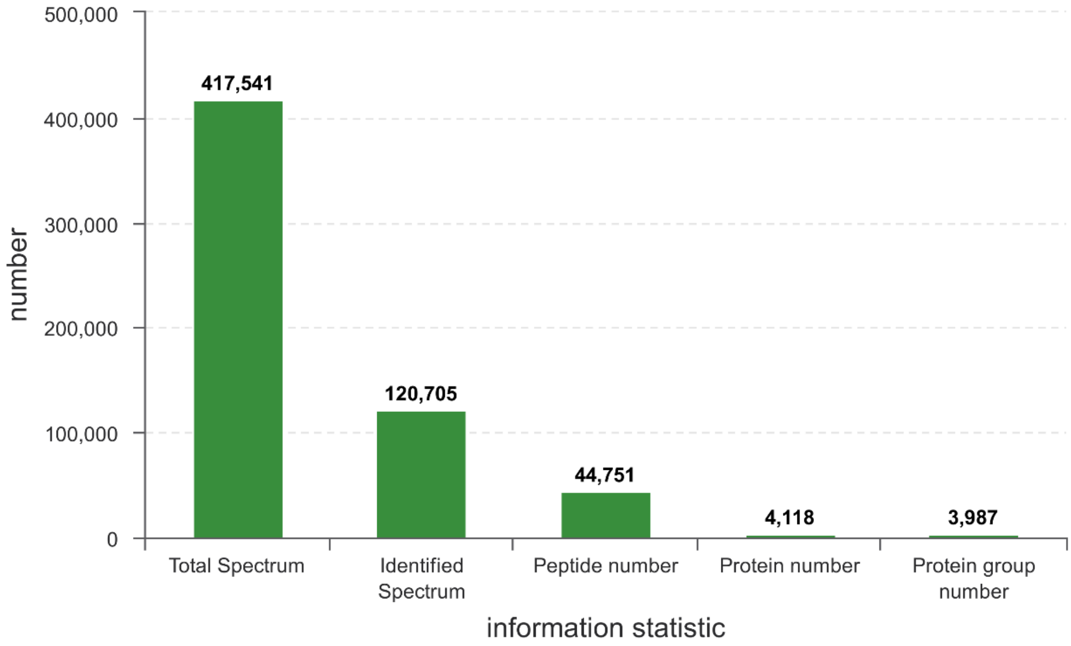

Fig. S4 A summary of protein information of *A. camphorata* S-29.

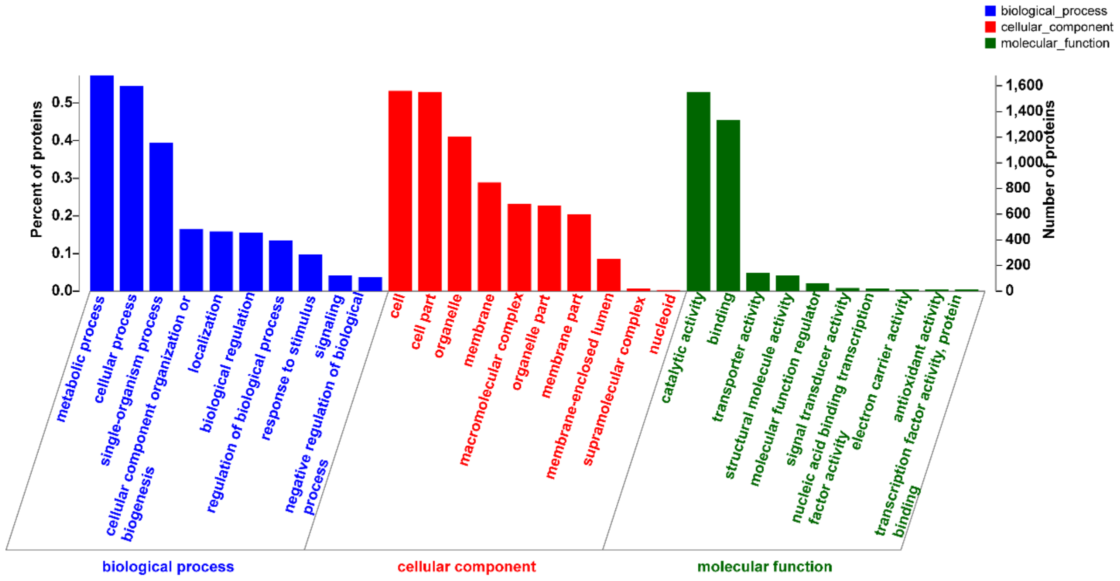

Fig. S5 The GO annotation of 3987 proteins in *A. camphorata* S-29.

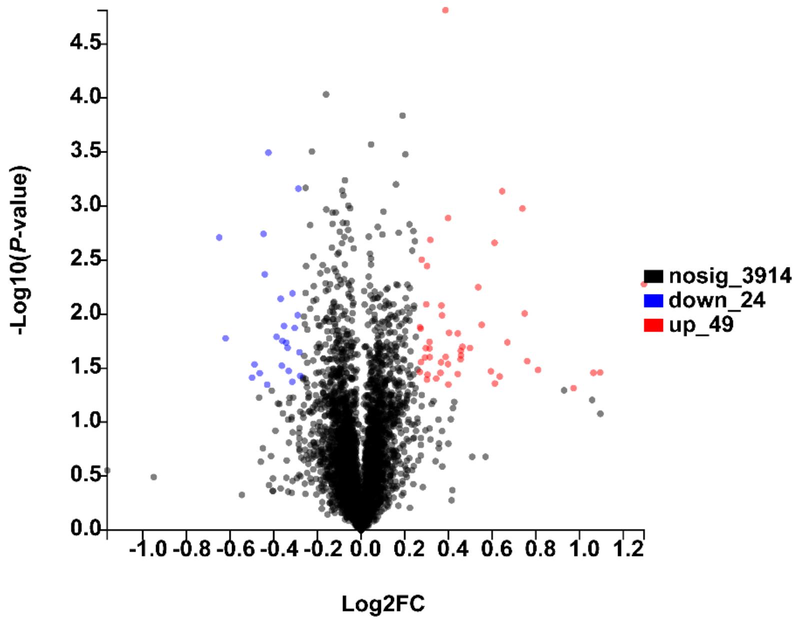

Fig. S6 Volcano plot of the 3987 proteins during FM compared with KB. Each point represents a specific protein, and the black points are the non-significant different proteins. The red dots indicate the significantly upregulated proteins, and the blue dots indicate the significantly downregulated proteins. The horizontal axis indicates fold change (FC) of gene expression. The vertical axis means the statistical significance level; the smaller the "*P*-value" is, the larger the "-log10 (*P*-value)" will be, which indicates that the difference is more significant.

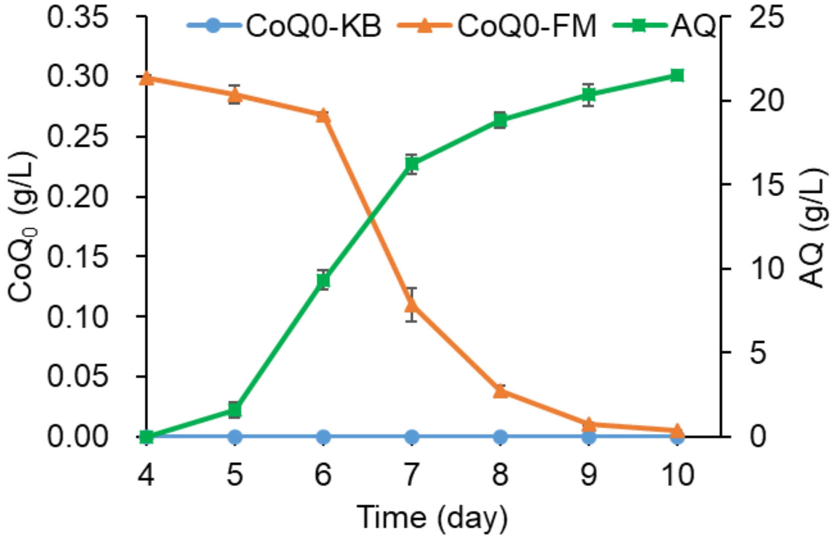

Fig. S7 The concentrations of CoQ_0 and AQ. The experiments were carried out with three replications. Values are given as the means ± standard deviations (n = 3).

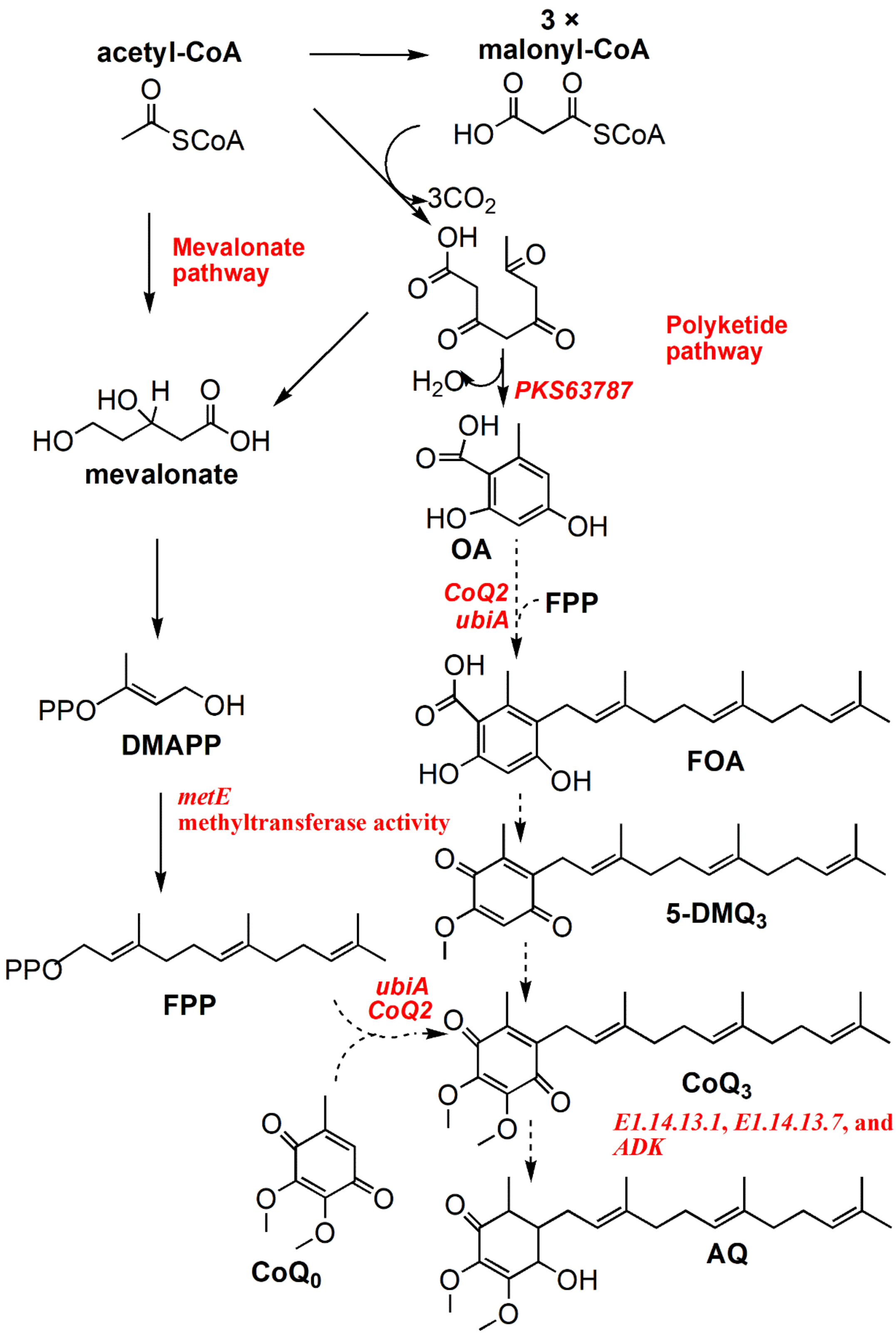

Fig. S8 Genes annotations in the AQ synthesis pathway during FM. FPP, farnesyl diphosphate; OA, orsellinic acid; FOA, 3-farnesyl-orsellinic acid; 5-DMQ3, 5-demethoxy- coenzyme Q_3; CoQ_3, coenzyme Q_3; AQ, antroquinonol.
